# Chemistry Related to the Catalytic Cycle of the Antioxidant Ebselen

**DOI:** 10.3390/molecules28093732

**Published:** 2023-04-26

**Authors:** Kai N. Sands, Austin L. Burman, Esther Ansah-Asamoah, Thomas G. Back

**Affiliations:** Department of Chemistry, University of Calgary, 2500 University Drive NW, Calgary, AB T2N 1N4, Canada; kai.sands@ucalgary.ca (K.N.S.); austinlukeburman@gmail.com (A.L.B.); estheraa@hotmail.co.uk (E.A.-A.)

**Keywords:** organoselenium chemistry, antioxidants, ebselen, seleninamide, seleninic acid, selenenyl sulfide, methanolysis, disproportionation

## Abstract

The antioxidant drug ebselen has been widely studied in both laboratories and in clinical trials. The catalytic mechanism by which it destroys hydrogen peroxide via reduction with glutathione or other thiols is complex and has been the subject of considerable debate. During reinvestigations of several key steps, we found that the seleninamide that comprises the first oxidation product of ebselen underwent facile reversible methanolysis to an unstable seleninate ester and two dimeric products. In its reaction with benzyl alcohol, the seleninamide produced a benzyl ester that reacted readily by selenoxide elimination, with formation of benzaldehyde. Oxidation of ebselen seleninic acid did not afford a selenonium seleninate salt as previously observed with benzene seleninic acid, but instead generated a mixture of the seleninic and selenonic acids. Thiolysis of ebselen with benzyl thiol was faster than oxidation by ca. an order of magnitude and produced a stable selenenyl sulfide. When glutathione was employed, the product rapidly disproportionated to glutathione disulfide and ebselen diselenide. Oxidation of the S-benzyl selenenyl sulfide, or thiolysis of the seleninamide with benzyl thiol, afforded a transient thiolseleninate that also readily underwent selenoxide elimination. The S-benzyl derivative disproportionated readily when catalyzed by the simultaneous presence of both the thiol and triethylamine. The phenylthio analogue disproportionated when exposed to ambient or UV (360 nm) light by a proposed radical mechanism. These observations provide additional insight into several reactions and intermediates related to ebselen.

## 1. Introduction

**Scheme 1:**
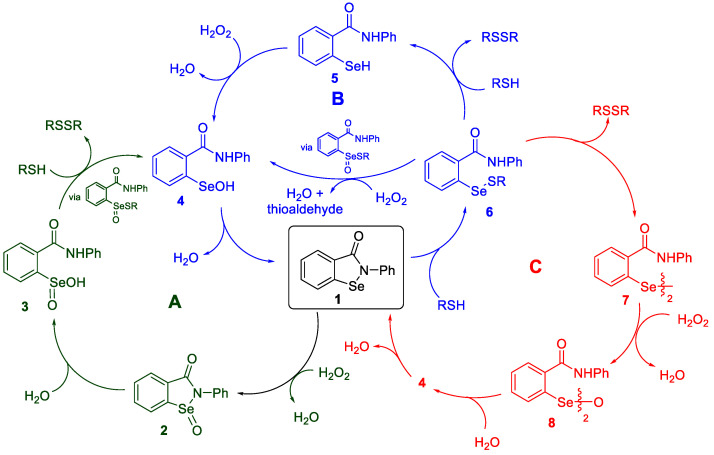
Pathways for the catalytic cycle of ebselen.

The glutathione peroxidases (GPx) act as biological antioxidants by catalytically destroying peroxides, formed during aerobic metabolism, with the tripeptide thiol glutathione (GSH) [1,2,3,4,5,6,7,8,9,10,11,12,13,14]. This ability serves to maintain redox balance in cells and minimizes oxidative stress caused by hydrogen peroxide, lipid hydroperoxides and downstream species such as the hydroxyl radical [15,16,17,18,19]. Several members of the GPx family owe their antioxidant activity to the presence of selenol moieties in the form of selenocysteine residues. Since oxidative stress has been implicated in many diseases and degenerative conditions, references [20,21,22,23,24,25,26,27] considerable effort has been directed toward the discovery of small-molecule mimetics of GPx that could be administered therapeutically [28,29,30,31,32,33,34,35,36,37,38,39]. Ebselen (**1**) has arguably been the most widely studied such selenium-based antioxidant. It was first reported by Lesser and Weiss in 1924 [40], but its antioxidant properties were not discovered until much later [41,42,43,44,45,46,47,48]. Numerous efforts to elucidate the catalytic cycle by which it reduces peroxides with stoichiometric amounts of thiols have been reported and several reviews on the subject have appeared [49,50,51,52]. Ebselen has undergone clinical trials as a neuro- and cardioprotective agent (Daiichi-Sankyo Inc. (Tokyo, Japan), Phase 3) [53,54], as a drug for restoring hearing loss (Sound Pharmaceuticals, Inc. (Seattle, WA, USA), Phase 2) [55,56] and in the treatment of bipolar disorder (Sound Pharmaceuticals, Inc. in collaboration with Oxford University (Oxford, UK), Phase 2) [57,58,59]. A clinical trial of ebselen as a therapy for SARS-CoV-2 was also announced recently [60]. Scheme 1 shows some of the key pathways that have been proposed in its catalytic antioxidant mechanism [61,62,63,64,65,66,67,68,69,70]. Since various groups have employed different thiols, peroxides, conditions and assay techniques in their mechanistic investigations, the results have not always been consistent. We have therefore re-examined several key steps and related intermediates in order to provide additional insight into their behavior and possible relevance to the overall catalytic process.

## 2. Results and Discussion

The first step in Scheme 1 (Scheme 1 is a modified and updated version of an earlier depiction of the possible mechanisms of ebselen [46]) can either be oxidation of ebselen by the peroxide to produce an oxidized intermediate such as **2** via (Path A) or thiolysis to afford the selenenyl sulfide **6** via (Path B) and its disproportionation product diselenide **7** (Path C). In principle, each of these intermediates **2** and **6**, along with the initial **1**, can be further transformed by either oxidation or reduction with the thiol, resulting in six key steps, which we investigated in turn.

### 2.1. Oxidation of Ebselen

**Scheme 2:**
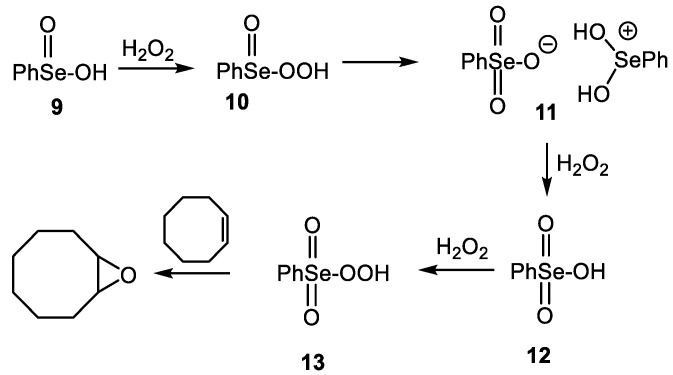
Intermediates in epoxidation with benzeneseleninic acid.

By comparison with ebselen, the oxidation of diphenyl diselenide and its congeners to afford the corresponding seleninic acids has been widely studied and the latter can be used as stoichiometric or catalytic oxidants for a variety of useful transformations (for selected reviews, see: [71,72,73,74,75,76]). We recently discovered that the epoxidation of cyclooctene effected with benzeneseleninic acid (**9**) in the presence of hydrogen peroxide proceeds chiefly through the corresponding peroxyselenonic acid **13 [77]** and not directly from the peroxyseleninic acid **10**, as previously suggested [78,79,80,81,82,83]. This is due to the rapid conversion of **10** to the selenonium selenonate salt **11**, followed by further oxidation to **12** and finally to **13** in the presence of hydrogen peroxide (Scheme 2). Several earlier reports of the preparation of **12 [84,85]** were subsequently shown to be in error, as the products proved to be the salt **11**, as later confirmed by x-ray crystallography [77]. It was therefore of interest to investigate the similar oxidation of ebselen to see whether products analogous to **11–13** would again be formed.

The oxidation of ebselen with hydrogen peroxide and other oxidants was reported to produce seleninamide **2** initially, which then underwent thiolysis to afford the selenenic acid **4** [62]. Furthermore, Kamigata et al. [86] reported that seleninamides were formed when various cyclic selenenamides related to ebselen were treated with ozone under anhydrous conditions, but the products were not characterized in full. Sarma and Mugesh [46] subsequently determined that oxidation of **1** with aqueous hydrogen peroxide produced the seleninic acid **3**, via the hydrolysis of initially formed **2**. The structure of **3** was unequivocally confirmed by x-ray crystallography.

**Scheme 3:**
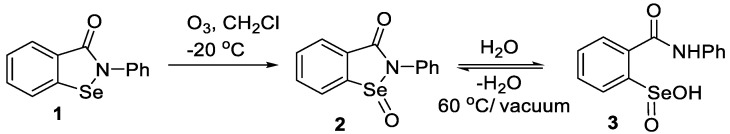
Oxidation and reversible hydrolysis of ebselen.

We re-examined the reaction of ebselen with ozone in anhydrous dichloromethane and isolated the seleninamide **2** in nearly quantitative yield. The product was thermally stable and was fully characterized but, as expected, was converted to the seleninic acid **3** upon exposure to water. When **3** was heated under vacuum, it regenerated the seleninamide **2** (Scheme 3). The reversibility of the hydration/dehydration of **2** and **3** was also evident during the preparation of **2** by oxidation of **1** with aqueous hydrogen peroxide in dichloromethane or chloroform. Under such conditions, the seleninamide **2** was isolated after 15 min, but on longer exposure to water (including water present in aqueous hydrogen peroxide), the main product was the seleninic acid **3**. These experiments confirm that the seleninamide can be formed initially by oxidation of **1**, but under physiological conditions, would undergo hydrolysis (or thiolysis, *vide infra*) to the seleninic acid.

It has also been suggested that further oxidation of seleninic acid **3** to the selenonic acid **14** might be feasible in excess hydrogen peroxide [67,70,87], but **14** has never been isolated or identified. Selenonic acids in general have been little studied, but a few have been reported from the further oxidation of the corresponding seleninic acids. Several examples of such oxidations were achieved by using potassium permanganate, followed by perchloric acid to liberate the free selenonic acids from their potassium salts [88,89,90], while several salts of carbohydrate-derived selenonic acids were obtained by using dimethyldioxirane (DMDO) as the oxidant [91]. The selenonic acid derivative of selenocysteine has also been prepared and its x-ray crystal structure was determined [92]. More recently, we described an effective one-pot synthesis of various selenonic acids from aryl bromides [93]. On the other hand, the formation of the isomeric peroxyseleninic acid **15** from **3** does not appear to have been reported during the oxidation of ebselen (Mugesh et al. [46] also postulated the formation of the corresponding peroxyselenenic acid intermediate (ArSe-OOH) from seleninic acid **3**. However, given the propensity for Se(II) species to undergo oxidation to Se(IV) in the presence of hydrogen peroxide and the expected greater stability of seleninic acid **3** compared to its peroxyselenenic acid isomer, this would likely comprise a very minor pathway).

**Figure 1:**
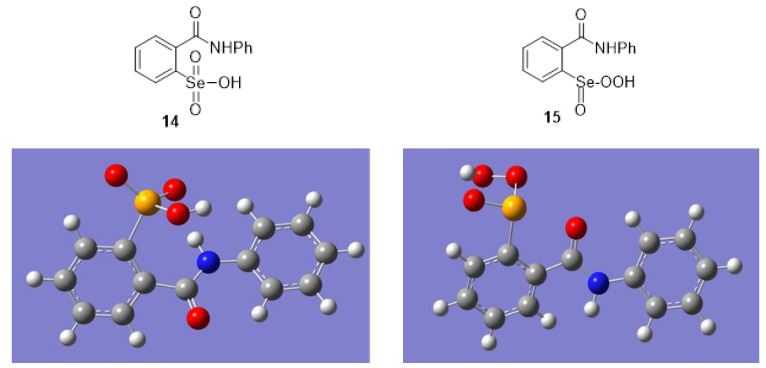
Geometry of optimized conformations of **14** and **15**.

In order to provide additional insight into the further oxidation of seleninic acid **3** during the oxidation of ebselen, we first performed Gaussian 09 DFT computations to obtain the optimized geometries and relative energies of the isomeric selenonic acid **14** and peroxyseleninic acid **15**, using the B3LYP platform, with cc-pVTZ and 6-311G(d,p) basis sets for Se and the lighter elements, respectively (for details, see the Supporting Information). Both **14** and **15** showed significant intramolecular hydrogen bonding between their hydroxyl or hydroperoxyl hydrogens and the corresponding selenoxide-like oxygen atoms, with OH---O distances of 2.576 Å and 2.026 Å, respectively. Peroxy acid **15** also displayed coordination between the carbonyl oxygen and selenium atoms, with a C=O---Se distance of 2.676 Å. On the other hand, **14** exhibited a strong hydrogen bond with a bond distance of 1.991 Å between the amide proton and a selenoxide oxygen atom (NH---O=Se). These interactions are within the sum of the van der Waals radii of the respective atoms. The two aryl moieties were not coplanar, exhibiting twist angles of 45.65° and 28.04° in **14** and **15**, respectively. The selenonic acid **14** proved more stable than the peroxyseleninic acid **15** by 28.4 kcal mol^−1^. The greater stability of **14** indicates that it would be the thermodynamically dominant product of the overall oxidation of seleninic acid **3.** An early report indicated that peroxyseleninic acid **10** isomerized to the selenonic acid 12 via a three-membered dioxyselenirane intermediate when heated in acetonitrile: [94]. However, subsequent work [77] revealed that the product was the salt **11** as indicated by x-ray crystallography. Interestingly, the conformation of **14** shown in Figure 1, where the carbonyl group is *anti* to the selenium moiety, is more stable than the *syn* conformer by 2.01 kcal mol^−1^. In contrast, the *syn* conformer of **15** shown in Figure 1 is more stable than the *anti* by 2.75 kcal mol^−1^.

**Figure 2:**
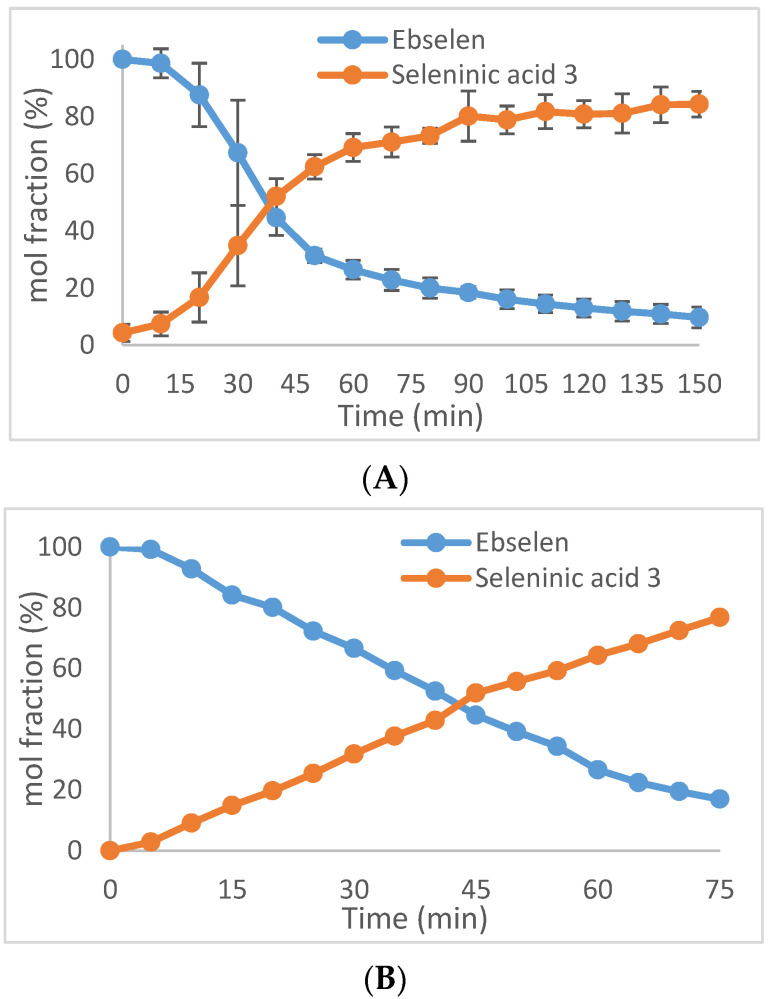
(**A**). Oxidation of ebselen (**1**) with 1.0 equiv of hydrogen peroxide. (**B**) Oxidation of ebselen (**1**) with 1.0 equiv of hydrogen peroxide and 0.1 equiv (10 mol %) of **3**. All reactions were conducted using 96 mg (0.35 mmol of ebselen in 10 mL of CDCl_3_ containing dimethyl sulfone (Me_2_SO_2_) as an internal standard). Error bars in (**A**) are based on triplicate runs shown at ±1 standard deviation. A single run was made in (**B**). The indicated mol % of **3** in (**B**) was adjusted for the 10% added at the start.

As shown in Scheme 1, the initial step in the reaction of ebselen in the presence of thiols and hydrogen peroxide (as in most assays or in vivo), can in principle be either oxidation to **2** or thiolysis to selenenyl sulfide **6**. In a separate experiment to establish relative rates of these processes, the oxidation of **1** with 1.0 equiv of hydrogen peroxide was monitored by ^1^H NMR spectroscopy in CDCl_3_ solution at 25 °C, revealing that the oxidation to **3** was 50% complete after 40 min and 90% complete after 140 min (Figure 2A). The oxidation affords the seleninamide **2** initially, followed by its hydrolysis to the seleninic acid, and is considerably slower than the thiolysis of **1** (vide infra) depicted in the catalytic cycles B and C of Scheme 1 and suggests that cycle A plays a minor role, if any, in the catalytic mechanism of ebselen under these conditions. This is in agreement with computational work by Antony and Bayse [95], who reported that the thiolysis of ebselen to the corresponding selenenyl sulfide is favored over its oxidation to the seleninamide **2**. The dominance of thiolysis over oxidation is expected to be even greater in vivo as a result of the high concentrations of glutathione and other thiols in cells, compared to the concentrations of ROS species. The brief induction period shown in Figure 2A suggests that autocatalysis by the product seleninic acid **3** in the oxidation of ebselen with hydrogen peroxide may be occurring, and the induction period was slightly but noticeably shortened by the addition of a catalytic amount of seleninic acid **3** at the start of the reaction. The linear nature of the plots in Figure 2B is also consistent with a catalytic reaction where the concentration of the active catalyst is constant.

**Scheme 4:**
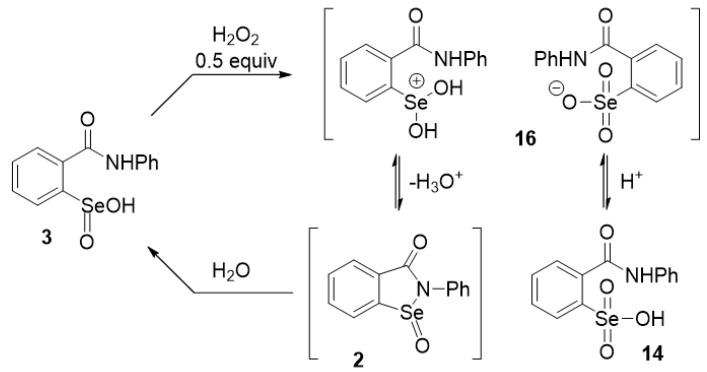
Oxidation of seleninic acid **3** with hydrogen peroxide.

It was also of interest to determine if the selenonium selenonate salt **16** was produced along the oxidation pathway leading from seleninic acid **3**, as was previously observed with **11** in the phenyl series shown in Scheme 2. By analogy, the reaction of seleninic acid **3** with 0.5 equiv of hydrogen peroxide was expected to produce salt **16** by conversion of 50% of **3** to selenonic acid **14**, followed by protonation of the remaining **3** by the stronger acid **14**. While the pKa’s of **3** and **14** have not been reported, the analogous pKa’s of benzeneseleninic and benzeneselenonic acids **9** and **12** were found to be 4.56 and 1.82, respectively [77]. An earlier pKa measurement of **9**, was reported as 4.79 [96]. The amphoteric nature of **9** has also been noted previously; see: [97]. Unfortunately, the process proved to be more complex than the one shown in Scheme 2 and we were unable to isolate pure **16** under a variety of conditions. Mixtures of products were obtained with their composition varying with solvent, duration of reaction, ratio of reactants and other conditions. The cleanest results were obtained when the reaction was performed in acetonitrile with 0.5 equiv of hydrogen peroxide, resulting in the formation of a white precipitate that displayed two faint peaks in the UDEFT ^77^Se NMR spectrum (The UDEFT protocol is useful for decreasing acquisition times for NMR signals from nuclei with slow relaxation times that are otherwise difficult to obsrve; see: [98]) at 1162 and 1020 ppm in DMSO-d_6_ (see Supporting Information). Longer acquisition times to obtain better ^77^Se spectra were precluded by the decomposition of the initial product. By comparison, an authentic sample of **3** gave a peak at 1143.3 ppm in DMSO-d_6_, which shifted to 1167.4 ppm with the addition of 1% D_2_O. This correlates closely with the downfield signal at 1162.5 ppm in the precipitate. In our previous work [93], all of the selenonic acids studied produced ^77^Se NMR signals in the 1020–1031 ppm range in D_2_O, consistent with the more upfield signal at 1020.2 ppm. These results suggest that the species present are the free seleninic and selenonic acids **3** and **14**, respectively, and not the expected salt **16**. The difference between this oxidation and that of benzeneseleninic acid **9** in Scheme 2 is likely due to the cyclization of the selenonium moiety of **16** with the amide side chain to produce **2**. In the presence of water, **2** is expected to hydrolyze to **3**, resulting in its regeneration (Scheme 4). Interestingly, oxidation of **3** with a full equivalent of hydrogen peroxide under a variety of conditions afforded mixtures in which the seleninic acid was the only identifiable product. Selenonic acid **14**, which to our knowledge has not yet been reported, could not be isolated from the oxidation of **3**. Longer reaction times in the oxidation resulted in intractable mixtures that were not investigated further. It therefore appears that the oxidation of ebselen seleninic acid **3** behaves quite differently from that of the phenyl analogue **9**, which can be attributed to the presence of the nucleophilic amido side chain in **3**.

### 2.2. Formation of Methyl Seleninates from Seleninic Acid ***3***

**Scheme 5:**
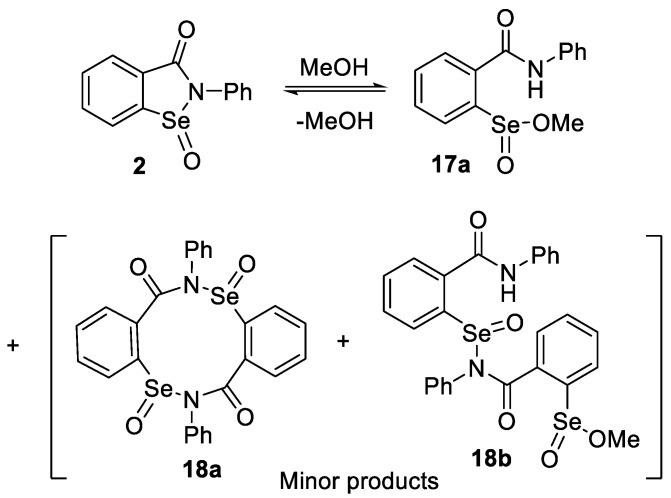
Methanolysis of seleninamide **2**.

**Scheme 6:**
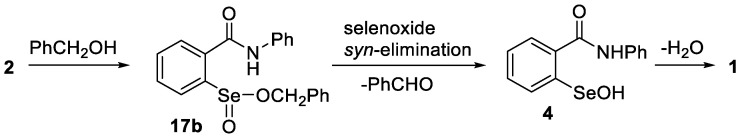
Reaction of seleninamide **2** with benzyl alcohol.

Due to the relatively poor aqueous solubility of ebselen and many other GPx mimetics, assays for measuring their relative catalytic activities in the reduction of hydrogen peroxide with thiols have frequently been performed in methanol or mixtures of methanol with other solvents. This raises the possibility that in the presence of the alcohol, methanolysis of oxidized ebselen species such as seleninamide **2** or esterification of seleninic acid **3**, which to our knowledge have not been previously reported, might compete with hydrolysis or other reactions of **2** in such assays. Indeed, when **2** was simply dissolved in methanol, the methyl ester **17a** was identified in the reaction mixture by NMR spectroscopy and ESI HRMS, along with the seleninamide **2**. Minor amounts of unexpected dimeric species consistent with structures **18a** and **18b** were also detected by HRMS (see Scheme 5 and Supporting Information for NMR and mass spectral data). Attempts to isolate the methyl ester **17a** by removal of methanol under vacuum afforded only **2**, identical to an authentic sample. These results indicate that the methyl ester **17a** and seleninamide **2** are in equilibrium in methanol solution and that removal of the solvent shifts the equilibrium to the cyclized product **2**. Similarly, **18a** and **18b** could not be isolated. Further evidence for the ability of seleninamide **2** to undergo spontaneous esterifications was obtained from its reaction with benzyl alcohol instead of methanol. After 3 h, the formation of benzaldehyde was observed in 20% yield (^1^H NMR), consistent with the formation of the benzyl ester **17b**, followed by a selenoxide *syn*-elimination to afford selenenic acid **4** and the aldehyde, and then cyclization and dehydration of **4** to ebselen (**1**) (Scheme 6). Prolonged exposure of **2** to methanol for 24 h also resulted in the generation of **1**, indicating that the methyl ester **17a** is formed and undergoes a similar *syn*-elimination, albeit more slowly than in the case of **17b**. These experiments indicate that a separate catalytic cycle under oxidative conditions in alcohols can operate via oxidation of **1**, followed by esterification of **2**, selenoxide elimination of **17** and cyclization/dehydration back to **1**.

While it is not known what precise effect the above processes have on the outcome of assays for GPx-like activity when conducted in methanolic media, the possibility exists that the results could be affected by esterification side reactions.

### 2.3. Thiolysis of Ebselen

**Figure 3:**
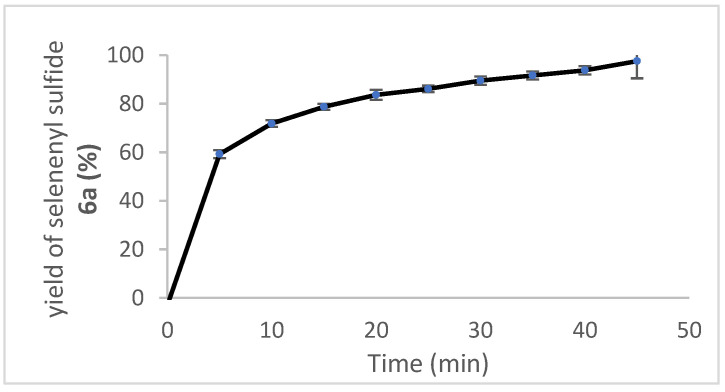
Thiolysis of ebselen with one equiv of benzyl thiol. The reaction was conducted using 96 mg (0.35 mmol) of ebselen in 10 mL of CDCl_3_ containing dimethyl sulfone (Me_2_SO_2_) as an internal standard. Reactions were initiated by the addition of 41 μL of BnSH (0.35 mmol). Analyses were in triplicate and error bars are shown at ±1 standard deviation.

**Figure 4:**
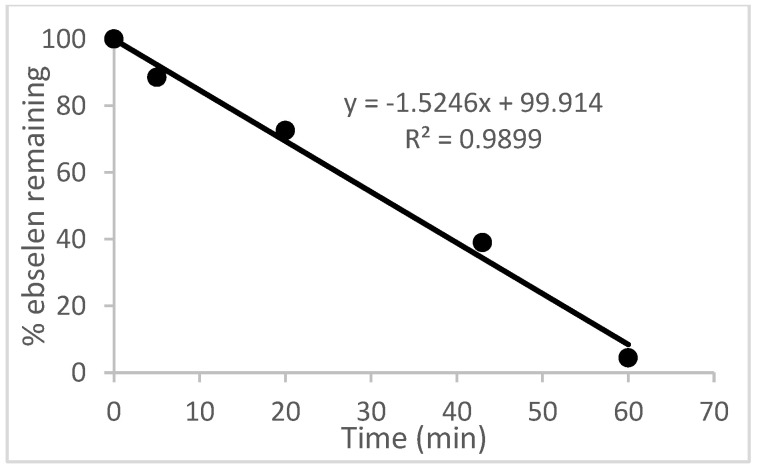
Thiolysis of ebselen with one equiv of glutathione under two-phase conditions. The reaction was conducted using 138 mg (0.503 mmol) of ebselen in 4 mL of 95:5 CDCl_3_/CD_3_OD containing dimethyl sulfone (Me_2_SO_2_) as an internal standard. Reactions were initiated by the addition of 153 mg (0.498 mmol) of glutathione in 1 mL of D_2_O with vigorous stirring to ensure proper mixing.

**Scheme 7:**
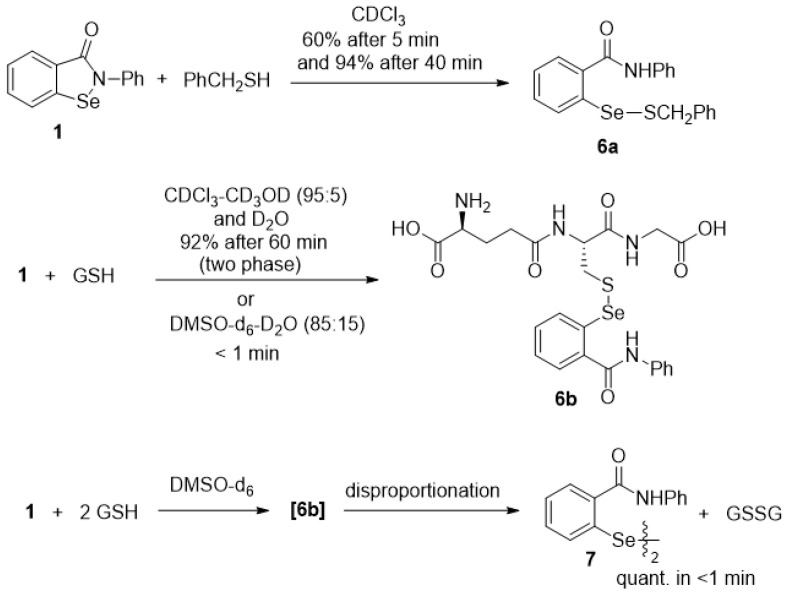
Thiolysis of ebselen.

In contrast to the relatively slow oxidation of ebselen (**1**) with hydrogen peroxide, its thiolysis with benzyl thiol in CDCl_3_ to produce the known selenenyl sulfide **6a [62]** was monitored by ^1^H NMR analysis, as shown in Figure 3. The reaction reached ca. 60% completion by the time the first data point was obtained after 5 min and was 94% complete after 40 min. The thiolysis of **1** was also performed with glutathione (GSH) in a two-phase system because of incompatible solubilities of the reactants, resulting in a slower reaction than with benzyl thiol. Thus, an aqueous solution of glutathione was added to an equimolar amount of **1** in CDCl_3_-CD_3_OD (95:5) with vigorous stirring, and the corresponding selenenyl sulfide **6b [63]** began to precipitate within a few minutes (Scheme 7). The disappearance of ebselen under these conditions was again monitored by ^1^H NMR analysis and is shown in Figure 4. The reason for the unexpectedly linear plot in Figure 4 is not clear at this time, but may be the result of concentration effects at the interface between the immiscible solvents where the reaction occurs. After 60 min, the precipitated selenenyl sulfide was collected in 92% yield. When the reaction was repeated in DMSO-d_6_/D_2_O (85:15) under homogeneous conditions, ebselen was consumed much more rapidly and the selenenyl sulfide **6b** was observed as the sole product within one min. Repetition of the above process with two equiv of glutathione resulted in almost immediate disproportionation of the initially formed selenenyl sulfide **6b** (vide infra) to the corresponding diselenide **7** and glutathione disulfide (GSSG). The entire process was again complete in less than 1 min, indicating that the disproportionation is accelerated by the presence of excess glutathione. These results are consistent with those reported by Mugesh et al. [69,70], who also noted that the rate of thiolysis of ebselen is highly dependent on the nature of the thiol.

These experiments show that the rate of thiolysis of **1** is considerably faster than its rate of oxidation (see Figure 2 for comparison) when performed under homogeneous conditions in either CDCl_3_ with benzyl thiol or in DMSO-d_6_ with glutathione. The faster rate of thiolysis compared to oxidation of ebselen confirms that the oxidative pathway A in Scheme 1 would only be competitive under extremely high concentrations of hydrogen peroxide and elevated conditions of peroxide-induced oxidative stress.

### 2.4. Reduction of Seleninamide ***2*** with Benzyl Thiol

Earlier studies of the reactions of benzeneseleninic acid (**9**) with *n*-butyl and *t*-butyl thiols were reported by Kice and Lee [99], who showed that thiolseleninates (RSSe(=O)Ph) are first generated, followed by their reaction with additional thiol to afford the corresponding selenenyl sulfides (RSSePh) and finally disproportionation to diphenyl diselenide and either di-*n*-butyl or di-*t*-butyl disulfide. With *t*-butyl thiol, the resulting thiolseleninate proved isolable, while the reaction of the *n*-butyl thiolseleninate with additional *n*-butyl thiol proved faster than its formation, thus precluding its direct observation. Kice and Purkiss [100] later showed that a radical process propagated by PhSeO• was implicated in the decomposition of the *t*-butyl thiolseleninate.

**Scheme 8:**
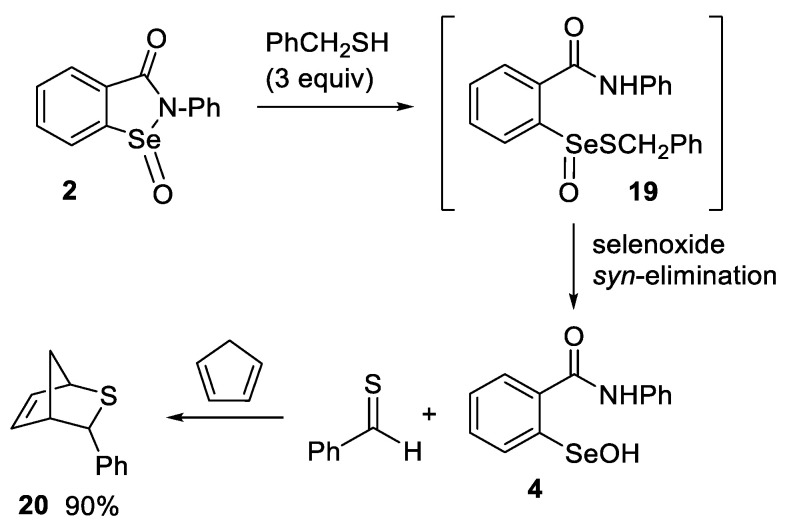
Trapping of thioaldehyde from the thiolysis and selenoxide elimination of **2** by Glass et al. [101].

The reaction of seleninamide **2** with benzyl thiol was investigated by Glass et al. in 1989 [101], but since then, this reaction has received limited attention. These researchers postulated that the thiolseleninate **19** was formed initially and then underwent a selenoxide elimination to produce thiobenzaldehyde and selenenic acid **4** (Scheme 8). The formation of the thiobenzaldehyde was confirmed by a trapping experiment with cyclopentadiene, which afforded the corresponding hetero-Diels-Alder cycloadduct **20** in 90% yield. The selenoxide elimination of another thiolseleninate had been previously reported by Reich et al. [102].

**Scheme 9:**
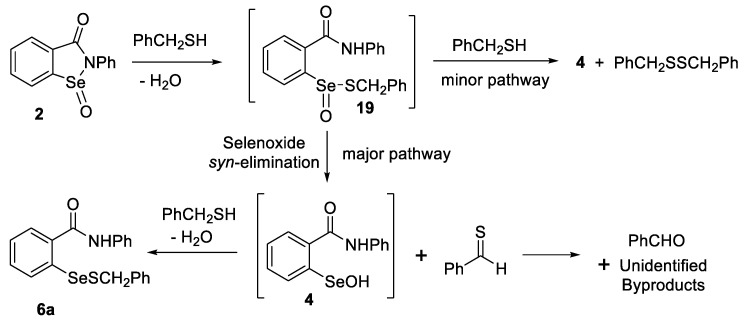
Pathways for thiolysis of seleninamide **2**.

We investigated the similar reaction of seleninamide **2** with three equiv of benzyl thiol under similar conditions to those of Glass et al. Instead of trapping the thioaldehyde produced by selenoxide elimination, the reaction mixture was analyzed by ^1^H NMR spectroscopy in CDCl_3_ with dimethyl sulfone as an internal standard. The reaction was essentially complete within 5 min and conversion of **3** to the selenenyl sulfide **6a** was observed, along with minor amounts of dibenzyl disulfide, similarly to the report by Glass et. al. Small amounts of benzaldehyde (^1^H NMR signal at 10.02 ppm) were also present, presumably from oxidation or hydrolysis of thiobenzaldehyde as well as one equiv of unreacted thiol. This supports the mechanism in Scheme 9 as the dominant pathway, in agreement with the previous work and consistent with the consumption of only two equiv of thiol. When only one equiv of benzyl thiol was employed, a transient blue colour characteristic of the thiolaldehyde formed immediately but faded within a few seconds. Similarly, the appearance of a faint AB quartet at 4.5 ppm in the ^1^H NMR spectrum of the reaction mixture is consistent with the transient formation of the chiral thiolseleninate **19**. The reaction of seleninamide **2** with either one or two equiv of benzyl thiol afforded more complex mixtures, including several unidentified selenium species that were present in the ^77^Se NMR spectra. The formation of dibenzyl disulfide can be rationalized by attack of the thiol on the sulfur atom of **19** in a minor pathway that would also lead to selenenic acid **4**. (Glass et al. [101] suggested that the disulfide could be produced by the reaction of selenenic acid **4** with thiobenzaldehyde and benzyl thiol). Moreover, the present reaction was too fast for rate measurement by NMR spectroscopy, since completion was essentially reached by the time the second spectrum could be acquired. It therefore appears that in the case of the unlikely formation of the seleninamide or seleninic acid in vivo (*vide supra*), it would have a very limited lifetime, given the abundance of glutathione and other native thiols in cells and the fast reaction rate observed in this model system.

### 2.5. Oxidation of Selenenyl Sulfides ***6a***

Since selenenyl sulfides **6** are relatively stable and are postulated to act as key intermediates in the mechanisms shown in paths B and C in Scheme 1, as well as comprising the principal product in Scheme 9, it was of interest to investigate their further transformations in more detail. Thus, the oxidation of **6a** with hydrogen peroxide in the absence of thiol was expected to generate the corresponding thiolseleninate **19** initially, followed by selenoxide elimination as in Scheme 9.

**Scheme 10:**
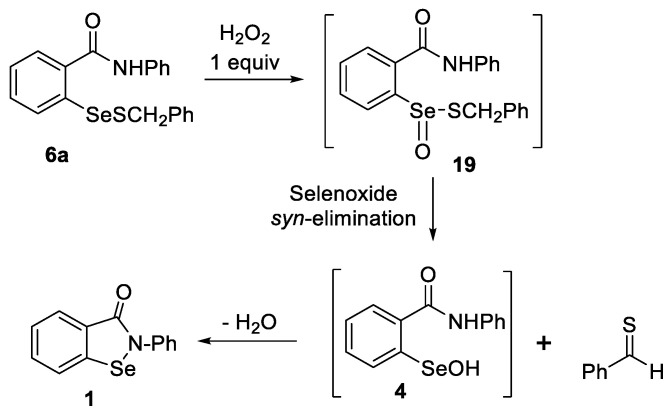
Oxidation of selenenyl sulfide **6a** with hydrogen peroxide.

The oxidation of **6a** with one equiv of hydrogen peroxide in CDCl_3_ was monitored by ^1^H NMR spectroscopy (Scheme 10 and Supporting Information). The consumption of **6a** reached 50% completion in 45 min, but the presence of 25% of unreacted **6a** persisted even after 100 min. Small amounts of dibenzyl disulfide were also formed, along with the highly insoluble ebselen diselenide **7**, which precipitated from the reaction mixture, presumably from the disproportionation of **6a**. Upon completion, the ^77^Se NMR spectrum revealed two signals at 961 and 560 ppm, matching those of ebselen and selenenyl sulfide **6a**, respectively (Scheme 10). Since the accumulation of significant amounts of thiolseleninate **19** was not observed, we conclude that the initial oxidation of **6a** is relatively slow compared to the more facile selenoxide elimination of thiolseleninate **19** or its decomposition via other pathways. As in Scheme 9, a ^1^H-NMR signal at 10.02 ppm was attributed to the formation of benzaldehyde from the hydrolysis or further oxidation of thiobenzaldehyde. When the reaction was repeated with 1.15 equiv of hydrogen peroxide in chloroform–methanol (95:5), ebselen was isolated in 82% yield.

As seen in path B of Scheme 1, the further reaction of selenenyl sulfides **6** with thiols can proceed via attack at the sulfur atom of **6**, thus generating the selenol **5** and the corresponding disulfide. Alternatively, disproportionation of the selenenyl sulfide results in a mixture of ebselen diselenide **7** and the disulfide (path C in Scheme 1). The selenol mechanism in path B has been validated by several groups, including via trapping of the selenol with various electrophiles [63,65,66] and through computational experiments [103,104].

Furthermore, Mugesh et al. [45] have shown that thiol attack at the selenium atom of **6** is also possible, but results in an unproductive thiol exchange process. The presence of substituents that coordinate with the selenium atom of **6**, as well as the use of glutathione as the thiol, favor the formation of the selenol **5**. The disproportionation of ebselen-derived selenenyl sulfides **6** to the corresponding disulfides and diselenides has also been studied [31,46,67], as well as that of other selenenyl sulfides (for examples of the disproportionation of other selenenyl sulfides, see [105,106,107,108]).

**Scheme 11:**
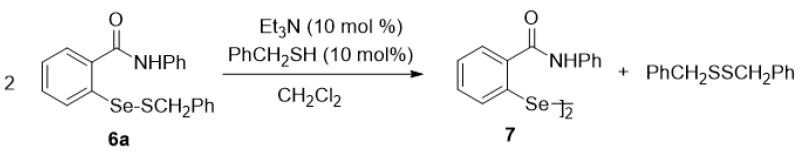
Disproportionation of selenenyl sulfide **6a**.

**Figure 5:**
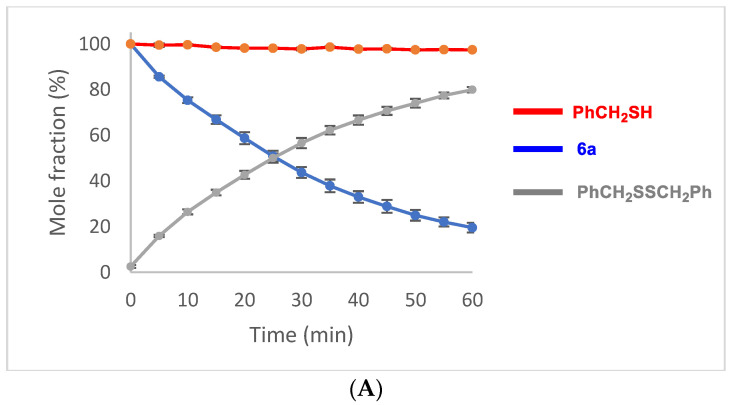

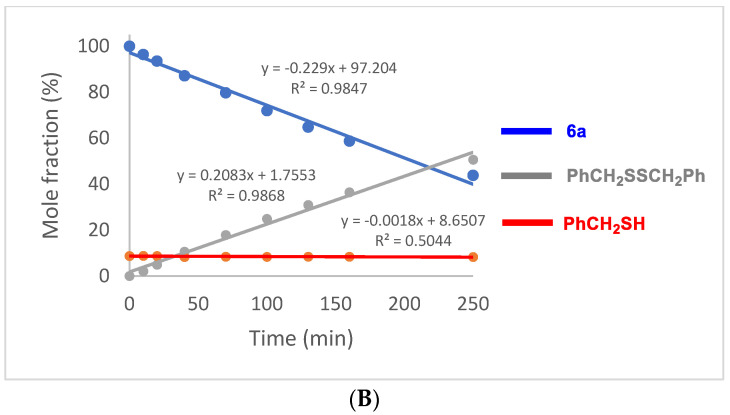
Disproportionation of selenenyl sulfide **6a** in the presence of (**A**) benzyl thiol (1 equiv) and triethylamine (10 mol %); (**B**) benzyl thiol 10 mol% and triethylamine (10%). Reactions in (**A**) were conducted using 139 mg (0.35 mmol) of **6a** in 10 mL of CDCl_3_ containing dimethyl sulfone (Me_2_SO_2_) as an internal standard. Reactions were initiated by the addition of 41 μL of BnSH (0.35 mmol) followed by 4.89 μL (0.035 mmol) of Et_3_N. In (**B**), only 0.035 mmol of BnSH was employed. Triplicate runs were made in (**A**) and error bars are shown at ±1 standard deviation. Only one run was recorded in (**B**).

**Scheme 12:**
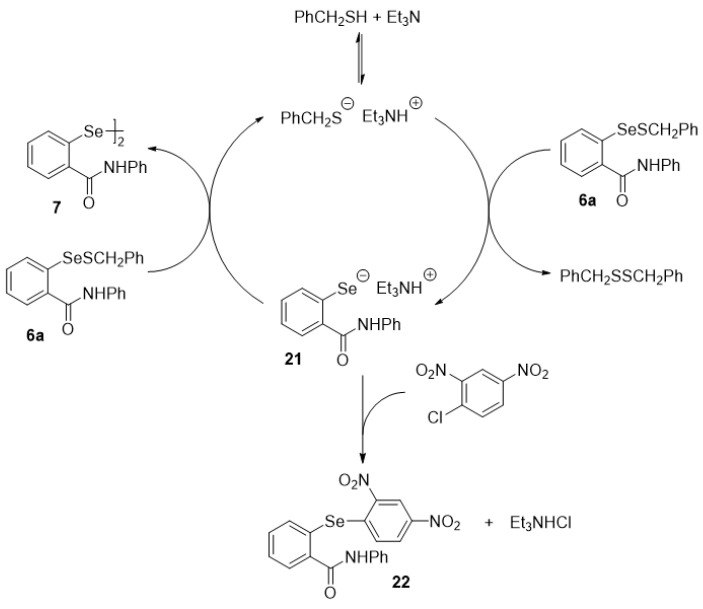
Catalytic mechanism for disproportionation of selenenyl sulfide **6a**.

In separate control experiments, we found that the benzyl derivative **6a** is stable in dichloromethane at room temperature, as well as in the presence of an equivalent amount of benzyl thiol, 10 mol % of trifluoroacetic acid, or 10 mol % of triethylamine. In each case, it was recovered unchanged after 28 h. However, in the presence of both 10 mol % of triethylamine and one equiv of benzyl thiol, the solution rapidly turned yellow and the highly insoluble diselenide **7** began to precipitate within 3 min. The disproportionation gave a t_1/2_ of 25 min and was essentially complete in 1 h, at which time filtration afforded **7** in 80% isolated yield (Scheme 11 and Figure 5). When only 10 mol % of the thiol was employed, the reaction still progressed to completion, albeit more slowly, with t_1/2_ = 220 min. The unchanging concentration of the thiol during these experiments confirms that the role of the thiol is catalytic. On the other hand, it will be recalled that the ebselen–glutathione selenenyl sulfide **6b**, in contrast to the benzyl analogue **6a**, underwent complete disproportionation within 1 min in DMSO-d_6_ in the presence of excess glutathione, as shown previously in Scheme 7. Presumably, excess glutathione provides both the catalytic thiol and amino groups required for the process. Based on these observations, we propose the process shown in Scheme 12 for the disproportionation of **6a**. The triethylamine is sufficiently basic to effect partial deprotonation of benzyl thiol (The pKa of both aliphatic thiols and of trialkylammonium species is ca. 10-11, thereby ensuring that about half of the thiol is deprotonated by an equimolar amount of triethylamine). The resulting thiolate reacts at the sulfur atom of **6a** to generate dibenzyl disulfide and the corresponding selenolate **21**, the presence of which was verified by trapping with 1-chloro-2,4-dinitrobenzene [65] to afford **22** in 98% yield. In the absence of the trapping agent, the selenolate then attacks a second molecule of **6a** at the selenium atom, thus producing diselenide **7** and regenerating the thiol catalyst.

**Figure 6:**
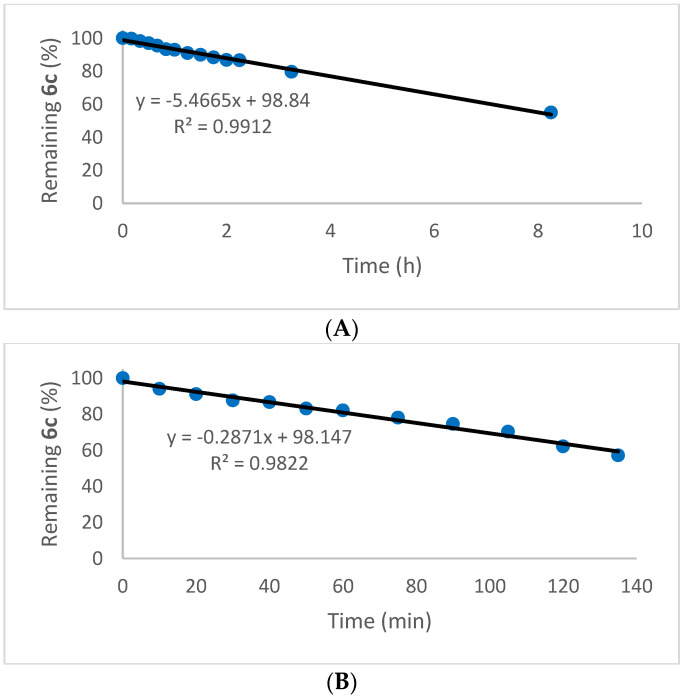
Photolytic disproportionation of selenenyl sulfide **6c**: (**A**) under ambient fluorescent lighting; (**B**) at 300 nm.

The photolysis of diselenides to produce selanyl radicals is well known [109,110], but does not appear to have been investigated in the case of selenenyl sulfides. While photochemical disproportionation of the latter is not relevant to biological antioxidant activity, it was nevertheless of interest to investigate the possibility. When a solution of selenenyl sulfide **6a** in deuterochloroform was exposed to ambient laboratory light or irradiated in a reactor containing 360 nm lamps, no disproportionation was evident, even after several days. However, when four 300 nm lamps were employed, disproportionation was observed with t_1/2_ = 24 h. When the latter reaction was repeated on a larger scale in dichloromethane for 12.5 h, the corresponding diselenide was isolated in 92% yield. The disproportionation of the selenenyl sulfide was driven to completion under either base-catalyzed or photochemical conditions by the poor solubility and precipitation of the diselenide. The photochemical disproportionation at 300 nm of the phenylthio analogue **6c** in CDCl_3_ behaved similarly, but at a faster rate, with 40% completion after 2.25 h, and even proceeded under ambient fluorescent laboratory lighting to 40% completion in 8 h (Figure 6). (The reason for the zero-order plots in Figure 6 is uncertain, but may be due to steady-state concentrations of excited states of the selenenyl sulfides). A control experiment where the phenylthio derivative in CDCl_3_ was kept in the dark at ambient temperature in the absence of added base and thiol showed no change even after two months. This confirms that light is essential for the disproportionation under these conditions and presumably occurs by homolysis of the S-Se bond, followed by radical substitution and recombination.

## 3. Summary and Conclusions

Each of the key steps proposed for the catalytic cycle of ebselen in the presence of hydrogen peroxide and either benzyl thiol or glutathione was investigated. Oxidation of ebselen proceeded via the seleninamide **2**, which was readily hydrolyzed to seleninic acid **3** and dehydrated back to **2** when heated under vacuum. The peroxyseleninic acid **15** was not observed and molecular modelling indicated that the isomeric selenonic acid **14** is considerably more stable, although it too was not isolated. The oxidation of ebselen was significantly slower than its thiolysis. Furthermore, the concentration of glutathione and of other thiols is typically much higher in cells than that of hydrogen peroxide, suggesting that Path A in Scheme 1 is unlikely under physiological conditions. The partial oxidation of **3** did not cleanly afford the selenonium selenonate salt **16**, as was previously observed with the formation of **11** in the phenyl series. Only the seleninic acid **3** and selenonic acid **14** could be identified as major constituents by NMR spectroscopy of the resulting complex mixtures. This suggests that, if formed, the selenonium ion of **16** undergoes cyclization to **2** and hydrolysis to **3** under these conditions. It was also observed that the oxidation of ebselen with hydrogen peroxide in methanol resulted in the formation of methyl esters of **3** as well as dimeric species. These esters are hydrolytically unstable and generate seleninamide **2** upon evaporation of methanol under vacuum. The esters also readily undergo selenoxide *syn*-elimination, followed by cyclization, to regenerate ebselen in a potentially competing catalytic cycle. This may have as yet undetermined implications for assay protocols used in the measurement of antioxidant activity that often employ methanol or CD_3_OD as the solvent or cosolvent.

The well-known thiolysis of ebselen affords the corresponding selenenyl sulfides. The rates of these processes vary with the thiol, but typically occur about one order of magnitude faster than the corresponding oxidation of ebselen with hydrogen peroxide. Interestingly, the use of excess glutathione resulted in very rapid disproportionation of the selenenyl sulfide to the corresponding diselenide and disulfide, attributed to the presence of both catalytic amino and thiol groups. The thiolysis of seleninamide **2** proved too rapid for rate measurement by NMR spectroscopy and required two equivalents of thiol to go to completion. A selenoxide elimination of an intermediate thiolsulfinate **19** was implicated, followed by thiolysis of the resulting selenenic acid **4** to produce selenenyl sulfide **6a**. When the thiolseleninate was generated by the oxidation of **6a** with one equivalent of hydrogen peroxide in the absence of thiol, the selenoxide elimination was again observed, but the product selenenic acid **4** cyclized to produce ebselen in high yield. Finally, the disproportionation of **6a** was investigated via a series of control reactions. The selenenyl sulfide remained intact when treated with either TFA, triethylamine or benzyl thiol. In contrast, the disproportionation was rapid in the simultaneous presence of catalytic amounts of both triethylamine and benzyl thiol. We conclude that a catalytic cycle involving the sequential generation of thiolate and selenolate anions takes place. Since glutathione contains both an amino and thiol group, it appears to catalyze the disproportionation even in the absence of an added amine. The disproportionation can also be effected photochemically, presumably by a radical mechanism. Although numerous mechanistic studies of ebselen have been previously reported, the present work thus adds additional insight into several key steps.

## 4. Materials and Methods

### 4.1. General Experimental

All reagents and starting materials were obtained from commercial sources and used without further purification unless otherwise noted. Tetrahydrofuran was dried over LiAlH_4_ and was freshly distilled before use. Dichloromethane was dried over anhydrous K_2_CO_3_ or distilled from CaH_2_. Hydrogen peroxide was titrated [111] before use and had a concentration of 50 ± 1%, or 29 ± 1%, unless otherwise indicated. The concentration of *n*-butyllithium was determined by titration with *N*-benzylbenzamide as the indicator [112]. Isolated yields are reported unless otherwise noted. Flash chromatography was performed using silica gel (230–400 mesh) as the stationary phase. ^1^H, ^13^C and ^77^Se NMR spectra were recorded in CDCl_3_, CD_3_CN, DMSO-d_6_, CD_3_OD, D_2_O or mixtures thereof as indicated, at 400 MHz, 101 MHz, 377 MHz or 76 MHz, respectively. ^1^H and ^13^C NMR spectra were referenced relative to the residual solvent (CHCl_3_: ^1^H at δ 7.26 ppm, ^13^C at δ 77.2 ppm; DMSO: ^1^H at δ 2.50 ppm, ^13^C at δ 39.5 ppm; acetonitrile: ^1^H at 1.94 ppm, ^13^C at δ 118.3 ppm; CD_3_OD: ^1^H at δ 3.31 ppm, ^13^C at δ 49.0 ppm; D_2_O: ^1^H at δ4.79 ppm). An external reference of Ph_2_Se_2_ (δ 463 relative to Me_2_Se) [113] was used for ^77^Se NMR spectra, respectively. ^13^C and ^77^Se NMR spectra were recorded with broadband proton decoupling and the UDEFT pulse program, which accelerated the acquisition of signals from nuclei with long relaxation times [98]. Exact mass determinations were performed using positive or negative ion electrospray ionization (ESI), unless otherwise noted.

Caution: Although no explosions were observed during the course of this work, peroxyseleninic and peroxyselenonic acids comprise potential explosion hazards and should be treated with appropriate precautions.

### 4.2. Preparation of Ebselen *(**1**)*

Ebselen was prepared by the method of Engman and Hallberg [114]. Recrystallization from ethanol gave the pure product in 63% yield as a light yellow solid: mp 182–183 °C (lit. [114] mp 180–181 °C); ^1^H NMR (400 MHz, CDCl_3_) δ 8.13 (d, *J* = 7.6 Hz, 1H), 7.70–7.62 (m, 4H), 7.50–7.40 (m, 3H), 7.29 (t, *J* = 7.5 Hz, 1H); ^13^C NMR (101 MHz, CDCl_3_) δ 165.7, 139.1, 137.6, 132.5, 129.4, 129.3, 127.6, 126.7, 126.5, 125.4, 123.7; ^77^Se NMR (76 MHz, CDCl_3_) 959.9 (lit. [62] δ 959, DMF-d_7_).

### 4.3. Preparation of 2-Phenylbenzo[d][1,2]selenazol-3(2H)-one 1-Oxide *(**2**)*

Ebselen (55.2 mg, 0.201 mmol) was stirred in dry CDCl_3_ (passed through anhydrous K_2_CO_3_) and cooled to −20 °C. Ozone was introduced into the reaction mixture for 12 min, until the yellow color was completely discharged and TLC analysis showed the absence of the starting material. The solvent was removed in vacuo to give 58.1 mg (99%) of the title compound as a white solid: mp (sealed tube) 178–182 °C (dec) (lit. [86] mp 189–190 °C); ^1^H NMR (400 MHz, CDCl_3_) δ 8.15 (dd, *J* = 6.9, 1.9 Hz, 1H), 7.96 (dd, *J* = 6.9, 1.6 Hz, 1H), 7.86–7.79 (m, 2H), 7.55 (dd, *J* = 8.7, 1.5 Hz, 2H), 7.49 (t, *J* = 7.3 Hz, 2H), 7.42 (tt, *J* = 7.3, 1.2 Hz, 1H); ^13^C NMR (101 MHz, CDCl_3_) δ 166.9, 135.8, 134.6, 133.2, 130.5, 129.7, 129.0, 128.5, 127.0, 126.3, 115.0; ^77^Se NMR (76 MHz, CDCl_3_) δ 1090.1; HRMS calcd. for C_13_H_9_NO_2_^80^Se: 291.9877 [M + H]^+^; found: 291.9878.

### 4.4. Preparation of 2-(Phenylcarbamoyl)benzeneseleninic Acid *(**3**)*

Ebselen (550 mg, 2.01 mmol) was dissolved in 40 mL of dichloromethane. Hydrogen peroxide (26.5%, 0.28 mL, 2.4 mmol) was added and the reaction was stirred at room temperature overnight. The resulting precipitate was collected by vacuum filtration, washed with cold dichloromethane and dried in vacuo to give 441 mg (71%) of seleninic acid **3** as an off-white solid: mp 186–189 °C (dec.); ^1^H NMR (400 MHz, DMSO-d_6_) δ 8.26 (d, *J* = 7.4 Hz, 1H), 7.96 (d, *J* = 7.4 Hz, 1H), 7.90 (t, *J* = 7.5 Hz, 1H) 7.82 (t, *J* = 7.5 Hz, 1.0 Hz, 1H), 7.50 (broad s, 4H), 7.41–7.34 (m, 1H); ^13^C NMR (101 MHz, DMSO-d_6_) δ 166.9, 148.2, 137.5, 134.6, 132.8, 131.6, 129.7, 127.9, 127.6, 126.9; ^77^Se NMR (76 MHz, DMSO-d_6_) δ 1143.3 (lit. [115] δ 1144 ppm, CD_3_OD); ^77^Se NMR (76 MHz, CDCl_3_) δ 1125.4 (lit. [46] δ 1122, CDCl_3_). The possibility that the seleninic acid **3** could oxidize the DMSO-d6 solvent under these conditions was ruled out by a control experiment in which nondeuterated DMSO in CDCl_3_ was exposed to **3** and showed no detectable amount of dimethyl sulfone after one hour. When hydrogen peroxide was added to the mixture, sulfoxide to sulfone oxidation was observed.

### 4.5. Preparation of 2-[(Benzylthio)selanyl]-N-phenylbenzamide *(**6a**)*

Ebselen (1.00 g, 3.65 mmol) was dissolved in dichloromethane (70 mL), and benzyl thiol (0.43 mL, 3.7 mmol) was added. The reaction mixture was stirred at room temperature for 3 h and volatile material was removed in vacuo. The crude product was purified by flash chromatography with dichloromethane as eluent to give 1.44 g (99%) of selenenyl sulfide **6a** as a white solid: mp 128–130 °C (lit. [101] mp 128–129 °C) ^1^H NMR (400 MHz, CDCl_3_) δ 8.16 (dd, *J* = 8.1, 1.1 Hz, 1H), 7.85 (br s, 1H), 7.68–7.61 (m, 3H), 7.45–7.37 (m, 3H), 7.32–7.27 (m, 3H), 7.27–7.17 (m, 4H), 4.03 (s, 2H); ^13^C NMR (101 MHz, CDCl_3_): δ 166.0, 138.3, 137.3, 136.9, 131.9, 131.8, 129.2, 129.0, 128.8, 128.4, 127.2, 126.5, 125.9, 125.0, 120.5, 42.2. ^77^Se NMR (76 MHz, CDCl_3_) δ 561.3.

### 4.6. Preparation of 2-[(Glutathionyl)selanyl]-N-phenylbenzamide *(**6b**)*

Ebselen (303 mg, 1.11 mmol) was dissolved in 6 mL of dichloromethane–methanol (95:5). Glutathione (307 mg, 1.00 mmol) in 3 mL of deionized water was added dropwise over 5 min with vigorous stirring. After 40 min a white precipitate formed and was filtered, washed with cold dichloromethane, followed by deionized water and dried in vacuo to yield 558 mg (95%) of the glutathione selenenyl sulfide **6b** (mp: gradual dec from 190 ºC) (lit. [62]: mp 246 °C, dec); ^1^H NMR (400 MHz, DMSO-d_6_) δ 10.60 (s, 1H), 8.70 (t, *J* = 5.5 Hz, 1H), 8.50 (d, *J* = 8.3 Hz, 1H), 8.20 (d, *J* = 8.1, 1H), 8.13 (d, *J* = 7.9 Hz, 1H), 7.74 (d, *J* = 8.7 Hz, 2H), 7.63 (t, *J* = 8.2 Hz, 1H), 7.44 (t, *J* = 7.3 Hz, 1H), 7.37 (t, *J* = 7.6 Hz, 2H), 7.14 (t, *J* = 7.4 Hz, 1H), 4.43 (dt, *J* = 9.6, 4.4, 1H), 3.68 (dd, *J* = 19.1, 6.0 Hz, 1H), 3.70–2.70 (m and HOD), 2.30 (m, 2H), 1.93 (m, 1H), 1.83 (m, 1H); ^13^C NMR (101 MHz, DMSO-d_6_) δ 172.3, 171.4, 171.1, 170.9, 166.7, 138.9, 135.9, 132.8, 132.0, 129.3, 129.1, 127.8, 126.4, 124.7, 121.4, 53.8, 53.5, 41.7, 39.2, 31.9, 27.2; ^77^Se NMR (76 MHz, DMSO-d_6_) δ 547.1; HRMS calcd. for C_23_H_26_N_4_O_7_S^80^Se: 583.0760 [M + H]^+^; found: 583.0751.

### 4.7. Preparation of 2-[(Phenylthio)selanyl]-N-phenylbenzamide *(**6c**)*

Ebselen (2.00 g, 7.29 mmol), was dissolved in dichloromethane (150 mL) and thiophenol (0.75 mL, 7.3 mmol) was added. The reaction mixture was stirred at room temperature for 2.5 h. The volatile material was removed in vacuo and the crude product was purified by flash chromatography, eluting with 100% dichloromethane to give 1.96 g (70%) of selenenyl sulfide **6c** as a white solid: mp 100–102 °C (lit. [45] reported as oil); ^1^H NMR (400 MHz, CDCl_3_) δ 8.24 (dd, *J* = 8.1, 1.2 Hz, 1H), 7.92 (br s, 1H), 7.70 (dd, *J* = 7.8, 1.4 Hz, 1H), 7.62 (dd, *J* = 8.6, 1.2 Hz, 2H), 7.54–7.47 (m, 3H), 7.39 (dd, *J* = 8.5, 7.5 Hz, 2H), 7.34 (td, *J* = 7.5, 1.2 Hz, 1H), 7.26–7.08 (m, 4H); ^13^C NMR (101 MHz, CDCl_3_) δ 166.2, 137.9, 137.3, 136.7, 132.6, 131.5, 129.4, 129.1, 129.1, 129.0, 126.7, 126.3, 125.3, 120.8; ^77^Se NMR (76 MHz, CDCl_3_): δ 591.1. HRMS (ESI) *m*/*z* calcd for C_19_H_15_NOS^80^Se: 386.0112 [M − H]^−^; found: 386.0112.

### 4.8. Preparation of 2,2′-Diselenobis(benzanilide) *(**7**)*

Diselenide **7** was prepared by the method of Engman and Hallberg [114]. Benzanilide (1.00 g, 5.07 mmol) was dissolved in 30 mL of dry THF and cooled to 0 °C in an ice-water bath. *n-*Butyllithium (2.5 M, 4.1 mL, 10.2 mmol) was then added dropwise and the reaction mixture was stirred at 0 °C for 30 min. Elemental selenium (0.40 g, 5.07 mmol) was added in one portion and the reaction was stirred at 0 °C for an additional 30 min. The reaction mixture was then poured into a solution of K_3_Fe(CN)_6_ (1.70 g, 5.16 mmol) in water (100 mL). The resulting off-yellow precipitate was collected by vacuum filtration and washed with water. The crude product was recrystallized from 1,2-dichlorobenzene to afford 91% of the title compound as a light yellow solid: The crude product was recrystallized from 1,2-dichlorobenzene to afford 91% of the title compound as a light yellow solid: mp 262–264 °C (lit. mp [114] 256–257 °C); ^1^H NMR (400 MHz, DMSO-d_6_) δ 10.54 (br s, 2H), 7.95 (dd, *J* = 7.4, 1.5 Hz, 2H), 7.79 (dd, *J* = 8.1, 1.3 Hz, 2H), 7.77 (dd, *J* = 9.0, 1.4 Hz, 4H), 7.46 (dt, *J* = 7.4, 1.5 Hz, 2H), 7.42 (dd, *J* = 7.5, 1.5 Hz, 2H), 7.39 (t, *J* = 8.7 Hz, 4H), 7.15 (t, *J* = 7.4 Hz, 2H); ^13^C NMR (101 MHz, DMSO-d_6_) δ 166.8, 139.1, 134.3, 132.5, 132.4, 130.6, 129.2, 129.1, 126.9, 124.6, 121.0; ^77^Se NMR (76 MHz, DMSO-d_6_) δ 417.8.

### 4.9. General Procedure for Kinetic Experiments

The following procedure was employed, unless otherwise noted. Kinetic measurements were conducted in new 20 mL vials using disposable glass stir bars, with a constant stirring rate of 1200 rpm, unless otherwise noted. Reactions were performed in 10 mL of CDCl_3_ at room temperature. Each data point was acquired by removing 100 μL aliquots of the reaction mixture, diluting with 400 μL of CDCl_3_ and analyzing by ^1^H NMR spectroscopy. Concentrations were determined by integration of distinct peaks of the reactant and/or product against the signal of the internal standard (2.98 ppm for dimethyl sulfone).

### 4.10. Oxidation of Ebselen with Hydrogen Peroxide (See Figure 2)

Ebselen (97 mg, 0.35 mmol) was dissolved in 10 mL of CDCl_3_ containing dimethyl sulfone (6.58 mg, 0.070 mmol) as an internal standard. Hydrogen peroxide (30%, 36.0 μL, 0.35 mmol) was added and the solution was stirred for 160 min. Integration of the peak at 7.65 ppm from **1** and 7.90 ppm from **3** relative to that at 2.98 ppm from dimethyl sulfone was employed to measure the concentrations of **1** and **3**. The t_1/2_ was determined to be 40 min and the reaction was 90% complete after 140 min.

The preceding experiment was repeated, except that seleninic acid **3** (11 mg, 0.035 mmol) was included in the reaction mixture. The 10 min induction period in the absence of **3** (Figure 2A) was reduced to 5 min in its presence (Figure 2B). 

### 4.11. Further Oxidation of Seleninamide ***2*** with 0.5 Equiv of Hydrogen Peroxide

Seleninamide **2** (107 mg, 0.347 mmol) was dissolved in 1.5 mL of acetonitrile and hydrogen peroxide, (50%, 10.0 µL, 0.176 mmol) was added and the reaction was stirred vigorously for 40 min, resulting in the formation of 104 mg of a white precipitate that was filtered and dried. It displayed two faint peaks in the UDEFT ^77^Se NMR spectrum at 1162.4 and 1020.3 ppm in DMSO-d_6_. Longer acquisition times to obtain better ^77^Se spectra were precluded by the decomposition of the initial product.

### 4.12. Esterification of Seleninamide ***2*** with Methanol: Formation of Methyl 2-(Phenylcarbamoyl)benzeneseleninate *(**17a**)* and Its Dimers ***18a*** and ***18b***

Seleninamide **2** (15.1 mg) was dissolved in 0.5 mL of methanol and the solution was stirred at room temperature for 70 min. Excess methanol was removed under high vacuum to yield 16.5 mg of a clear oil. The ^1^H, ^13^C and ^77^Se NMR spectra indicated the presence of seleninamide **2** and a trace of residual methanol.

In a similar experiment, **2** (10.4 mg) was dissolved in 1 mL of dry CHCl_3_-MeOH (95:5) and the mixture was analyzed by ESI HRMS after standing at room temperature for 1 h. The following signals were observed: HRMS of **17a** calcd for C_14_H_13_NO_3_^80^Se: 324.0139 [M + H]^+^; found: 324.0130; HRMS of **18a** calcd for C_26_H_18_N_2_O_4_^80^Se_2_: 582.9675 [M + H]^+^; found: 582.9660; HRMS of **18b** calcd for C_27_H_22_N_2_O_5_^80^Se_2_: [M + H]^+^614.9937; found: 614.9922. (See Supporting Information for additional ^1^H NMR and mass specroscopic data).

Exposure of **2** to methanol for 24 h resulted in the formation of ebselen (**1**), identical to an authentic sample and consistent with a selenoxide elimination, such as the one shown in Scheme 6 for **17b**.

### 4.13. Esterification of Seleninamide ***2*** with Benzyl Alcohol

Seleninamide **2** (109 mg, 0.376 mmol) was dissolved in 9.5 mL of CDCl_3_ containing dimethyl sulfone (6.58 mg, 0.070 mmol) as an internal standard. Benzyl alcohol (0.50 mL, 4.8 mmol) was added and the solution was stirred at room temperature for 45 min, while monitored by ^1^H NMR spectroscopy. The formation of benzaldehyde (ca. 20%) was evident from the NMR signal at 10.02 ppm and NMR spectroscopy indicated that ebselen was the major product after removal of volatile material under vacuum.

### 4.14. Thiolysis of Ebselen with Benzyl Thiol (Figure 3)

Ebselen (96 mg, 0.35 mmol) was dissolved in 10 mL of CDCl_3_ containing dimethyl sulfone (6.58 mg, 0.070 mmol) as an internal standard. Benzyl thiol (41.0 μL, 0.35 mmol) was added and the mixture was stirred at 1200 rpm at room temperature for 45 min. Integration of the ^1^H NMR peak at 4.02 ppm from selenenyl sulfide **6a** relative to that at 2.98 ppm from dimethyl sulfone was employed to measure the concentration of **6a**. The reaction reached 59% completion by the time the first data point was collected (<5 min).

### 4.15. Thiolysis of Ebselen with Glutathione (Figure 4)

Ebselen (138 mg, 0.503 mmol) was dissolved in 4 mL of CDCl_3_-CD_3_OD (95:5) containing dimethyl sulfone (9.2 mg, 0.098 mmol) as an internal standard. Glutathione (153 mg, 0.498 mmol) dissolved in 1 mL of D_2_O was added and the mixture was stirred as vigorously as possible at room temperature for 60 min. A white precipitate began to form within 2 min after the addition of glutathione. Data points were acquired by briefly stopping the stirring to enable the layers to separate and taking 100 μL aliquots from the lower organic layer. Each aliquot was then diluted with 400 μL of CDCl_3_ and analyzed by ^1^H NMR spectroscopy. Integration of the peak at 8.14 ppm from **1** relative to that at 2.98 ppm from dimethyl sulfone was employed to measure the concentration of **1**. The reaction was complete in ca. 1 h. The concentration of selenenyl sulfide **6b** in the reaction mixture could not be determined due to its precipitation, but it was isolated at the end of the reaction by filtration, washing with water and dichloromethane, and drying in vacuo to afford a yield of 266 mg (92%).

When the reaction was repeated in DMSO-d_6_ -D_2_O (85:15) under homogeneous conditions, the formation of **6b** was complete in ca. 1 min. Repetition with two equiv of glutathione was equally rapid, but complete disproportionation of **6b** to diselenide **7** and glutathione disulfide was observed.

### 4.16. Reaction of Seleninamide ***2*** with Benzyl Thiol

Seleninamide **2** (109 mg, 0.375 mmol) was dissolved in 10 mL of CDCl_3_ containing dimethyl sulfone (6.58 mg, 0.07 mmol) as an internal standard. Benzyl thiol (123 μL, 1.05 mmol) was added and the mixture was stirred at room temperature for 40 min. The reaction was complete (<5 min) before the next spectrum could be acquired. It revealed the presence of one equiv of unreacted thiol along with one equiv of selenenyl sulfide **6a**. Minor amounts of benzaldehyde (10.02 ppm) and dibenzyl disulfide were also observed. The ^77^Se NMR spectrum showed a single peak at 535.4 ppm, indicative of **6a**. When the reaction was repeated with one or two equivs of benzyl thiol, more complex mixtures were obtained containing unidentified products.

### 4.17. Oxidation of Selenenyl Sulfide ***6a*** with Hydrogen Peroxide

Selenenyl sulfide **6a** (139 mg, 0.349 mmol) was dissolved in 10 mL of CDCl_3_ containing dimethyl sulfone (6.6 mg, 0.070 mmol) as an internal standard. Hydrogen peroxide (30%, 35.7 μL, 0.35 mmol) was added and the mixture was stirred at room temperature and monitored by ^1^H NMR spectroscopy for 60 min. The reaction reached 50% completion in 45 min, but 25% of unreacted **6a** persisted even after 100 min. Minor amounts of dibenzyl disulfide and benzaldehyde were also detected, along with a small amount of diselenide **7** that precipitated. In a separate reaction conducted with 1.1 equiv of hydrogen peroxide, ebselen was isolated in 82% yield by flash chromatography (eluent 100% dichloromethane—dichloromethane–methanol (9:1)), identical to an authentic sample.

### 4.18. Disproportionation of Selenenyl Sulfide ***6a***

*With benzyl thiol as additive*: Selenenyl sulfide **6a** (40.0 mg, 0.100 mmol) was dissolved in 2.0 mL of dry dichloromethane. Benzyl thiol (12 μL, 0.10 mmol) was added and the mixture was stirred at room temperature for 27.5 h. The reaction mixture was purified by flash chromatography using dichloromethane as eluent, affording 39.7 mg (99%) of recovered **6a**.

*With benzyl thiol and TFA as additives:* The reaction was repeated with **6a** (40.5 mg, 0.102 mmol) benzyl thiol (12 μL, 0.10 mmol) and 10 mol % of TFA (1 μL, 0.01 mmol) for 28 h. The selenenyl sulfide **6a** was recovered in 98% yield.

*With benzyl thiol and triethylamine as additives (*Figure 5*):* Selenenyl sulfide **6a** (139 mg, 0.349 mmol) was dissolved in 10 mL of CDCl_3_ containing dimethyl sulfone (6.6 mg, 0.070 mmol) as an internal standard. Benzyl thiol (41.0 μL, 0.349 mmol) was added, followed by triethylamine (4.89 μL, 0.035 mmol), and the mixture was stirred at room temperature and monitored by ^1^H NMR spectroscopy for 60 min. The consumption of **6a** and the formation of dibenzyl disulfide were 50% complete after 25 min, while the concentration of benzyl thiol remained unchanged. A white precipitate of diselenide **7** began to form within 3 min of the addition of triethylamine.

When the above reaction was repeated with only 10 mol % of benzyl thiol, the reaction was 50% complete in 220 min and there was again no change in the concentration of the thiol.

### 4.19. Trapping of Selenolate ***21*** [65]

Selenenyl sulfide **6a** (198 mg, 0.497 mmol) and 2,4-dinitrochlorobenzene (202 mg, 1.00 mmol) were dissolved in 10 mL of dichloromethane. Benzyl thiol (59 μL, 0.50 mmol) was added, followed by triethylamine (70 μL, 0.50 mmol). The solution immediately began to take on a deep yellow color. The mixture was stirred at room temperature for 2 h and concentrated in vacuo. The crude product was purified by flash chromatography, with 100% dichloromethane—10:1 dichloromethane–methanol as eluent to give 217 mg (98% of selenide **22** as a bright yellow solid: mp 175–180 °C (lit. [65] mp 177–178 °C); ^1^H NMR (400 MHz, CDCl_3_) δ 9.08 (d, *J* = 2.5 Hz, 1H), 8.12 (dd, *J* = 8.9, 2.5 Hz, 1H), 7.82 (t, *J* = 9.4 Hz, 1H), 7.69 (td, *J* = 7.6, 1.3 Hz, 1H), 7.59 (td, *J* = 7.6, 1.5 Hz, 2H), 7.47 (d, *J* = 7.3 Hz, 2H), 7.32 (t, *J* = 7.9 Hz, 2H), 7.28 (d, *J* = 9.0 Hz, 1H), 7.14 (t, *J* 6.9 Hz, 1H). ^13^C NMR (101 MHz, CDCl_3_): δ 166.4, 145.8, 145.4, 144.5, 143.1, 139.1, 137.3, 132.6, 132.2, 131.5, 129.4, 128.8, 127.0, 125.7, 125.3, 121.3, 120.2. ^77^Se NMR (76 MHz, CDCl_3_): δ 498.3. HRMS (ESI) *m*/*z* calcd for C_19_H_13_N_3_O_5_^80^Se: 465.9913 [M + Na]^+^; found: 465.9917. 

### 4.20. Photolytic Disproportionation of Selenenyl Sulfide ***6a***

Selenenyl sulfide **6a** (139 mg, 0.349 mmol) was dissolved in 10 mL of CDCl_3_ containing dimethyl sulfone (6.6 mg, 0.070 mmol) as an internal standard. The solution was irradiated at 300 nm (4 × 3.9 W lamps) for 24 h, at which time the yield of dibenzyl disulfide was ca. 50%.

### 4.21. Photolytic Disproportionation of Selenenyl Sulfide ***6c***

A sample of the phenylthio analogue **6c** in CDCl_3_ in an NMR tube wrapped in aluminum foil was kept in the dark at ambient temperature for two months. The ^1^H NMR spectrum remained unchanged.

When a similar sample was instead exposed to ambient fluorescent laboratory lighting for 8 h, ca. 40% underwent disproportionation. Irradiation at 300 nm for 135 min also resulted in ca. 40% disproportionation.

## Data Availability

Data supporting reported results can found on the *Molecules* web site or obtained by e-mailing T.G.B.

## Supplementary Materials

The following supporting information can be downloaded at https://www.mdpi.com/article/10.3390/molecules28093732/s1, Supporting Information: Figures S1–S29. Reference [116] is cited in the supplementary materials.
