# Supplementary material for: Chemistry Related to the Catalytic Cycle of the Antioxidant Ebselen
Source: Molecules. 2023 Apr 26;28(9):3732. doi: 10.3390/molecules28093732 (PMC10180093; doi:10.3390/molecules28093732)
Supplement: Supplementary file 1 [file molecules-28-03732-s001.zip › molecules-2352814-supplementary.pdf]

SUPPORTING INFORMATION

for

Chemistry Related to the Catalytic Cycle of the Antioxidant Ebselen

Kai N. Sands, Austin L. Burman, Esther Ansah-Asamoah and Thomas G. Back *

Department of Chemistry, University of Calgary, 2500 University Drive NW, Calgary, AB T2N 1N4, Canada

Contents

Figure S1. ^{1}H NMR Spectrum of Ebselen (**1**). p. 2
Figure S2. ^{13}C NMR Spectrum of Ebselen (**1**). p. 2
Figure S3. ^{77}Se NMR Spectrum of Ebselen (**1**). p. 3
Figure S4. ^{1}H NMR Spectrum of Seleninamide **2**. p. 3
Figure S5. ^{13}C NMR Spectrum of Seleninamide **2**. p. 4
Figure S6. ^{77}Se NMR Spectrum of Seleninamide **2**. p. 4
Figure S7. ^{1}H NMR Spectrum of Seleninic Acid **3**. p. 5
Figure S8. ^{13}C NMR Spectrum of Seleninic Acid **3**. p. 5
Figure S9. ^{77}Se NMR Spectrum of Seleninic Acid **3**. p. 6
Figure S10. ^{1}H NMR Spectrum of Selenenyl Sulfide **6a**. p. 6
Figure S11. ^{13}C NMR Spectrum of Selenenyl Sulfide **6a**. p. 7
Figure S12. ^{77}Se NMR Spectrum of Selenenyl Sulfide **6a**. p. 7
Figure S13. ^{1}H NMR Spectrum of Selenenyl Sulfide **6b**. p. 8
Figure S14. ^{13}C NMR Spectrum of Selenenyl Sulfide **6b**. p. 8
Figure S15. ^{77}Se NMR Spectrum of Selenenyl Sulfide **6b**. p. 9
Figure S16. ^{1}H NMR Spectrum of Selenenyl Sulfide **6c**. p. 9
Figure S17. ^{13}C NMR Spectrum of Selenenyl Sulfide **6c**. p. 10
Figure S18. ^{77}Se NMR Spectrum of Selenenyl Sulfide **6c**. p. 10
Figure S19. ^{1}H NMR Spectrum of Diselenide **7**. p.11
Figure S20. ^{13}C NMR Spectrum of Diselenide **7**. p.11
Figure S21. ^{77}Se NMR Spectrum of Diselenide **7**. p.12
Figure S22. ^{1}H NMR Spectrum of Selenide **22**. p. 12
Figure S23. ^{13}C NMR Spectrum of Selenide **22**. p. 13
Figure S24. ^{77}Se NMR Spectrum of Selenide **22**. p. 13
Figure S25. ^{77}Se NMR Spectrum of the Product of Oxidation of Seleninic Acid **3**. p. 14
Figure S26. Methanolysis of Seleninamide **2** by NMR spectroscopy. p. 15
Figure S27. Relative abundance of the $[M+H]^{+}$ peaks in the methanolysis of **2**. p. 16
Figure S28. Oxidation of Selenenyl Sulfide **6a** with Hydrogen Peroxide. p. 17
Figure S29. Molecular Modelling of **14** and **15**. p. 18

Figure S1. ^{1}H NMR Spectrum of Ebselen (1) (400 MHz, $CDCl_3$).

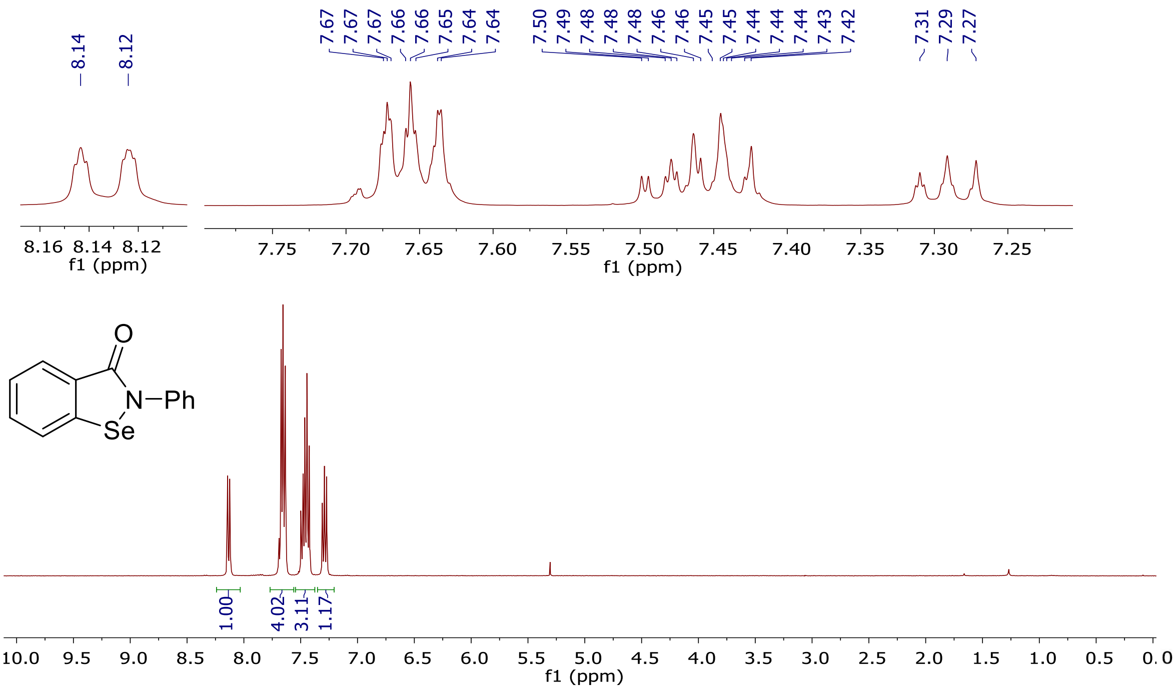

Figure S2. ^{13}C NMR Spectrum of Ebselen (1) (101 MHz, $CDCl_3$).

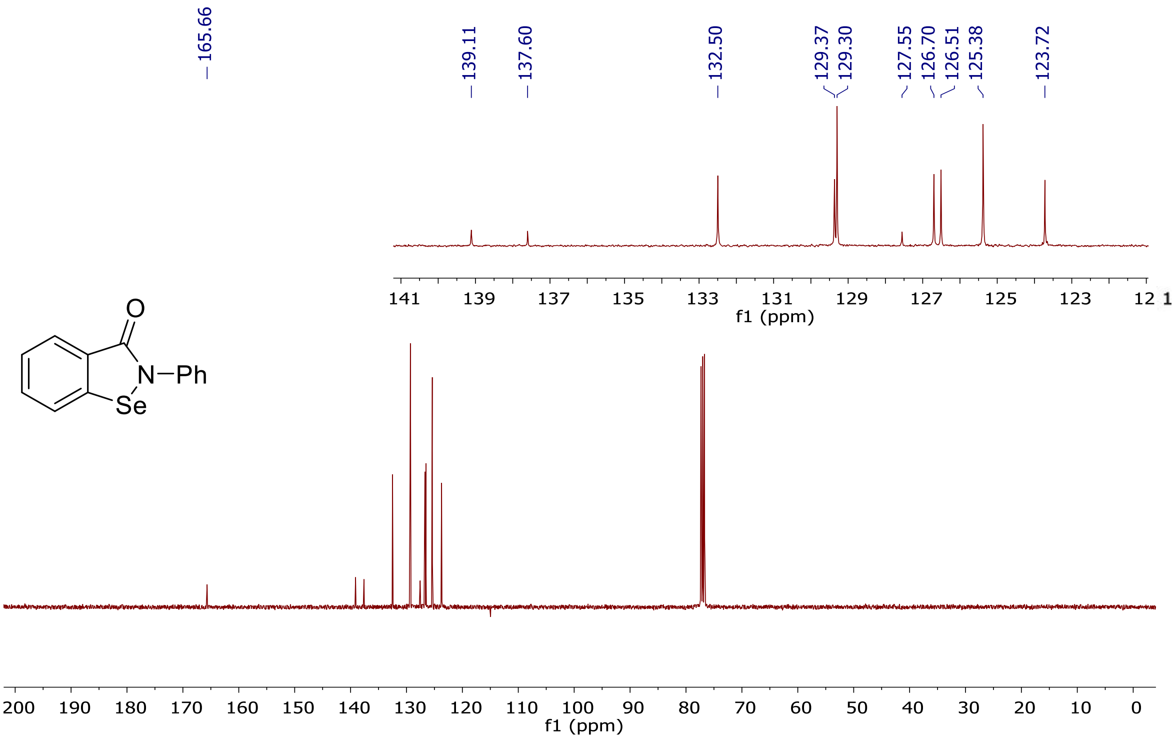

Figure S3. ^{77}Se NMR Spectrum of Ebselen (1) (76 MHz, $CDCl_3$).

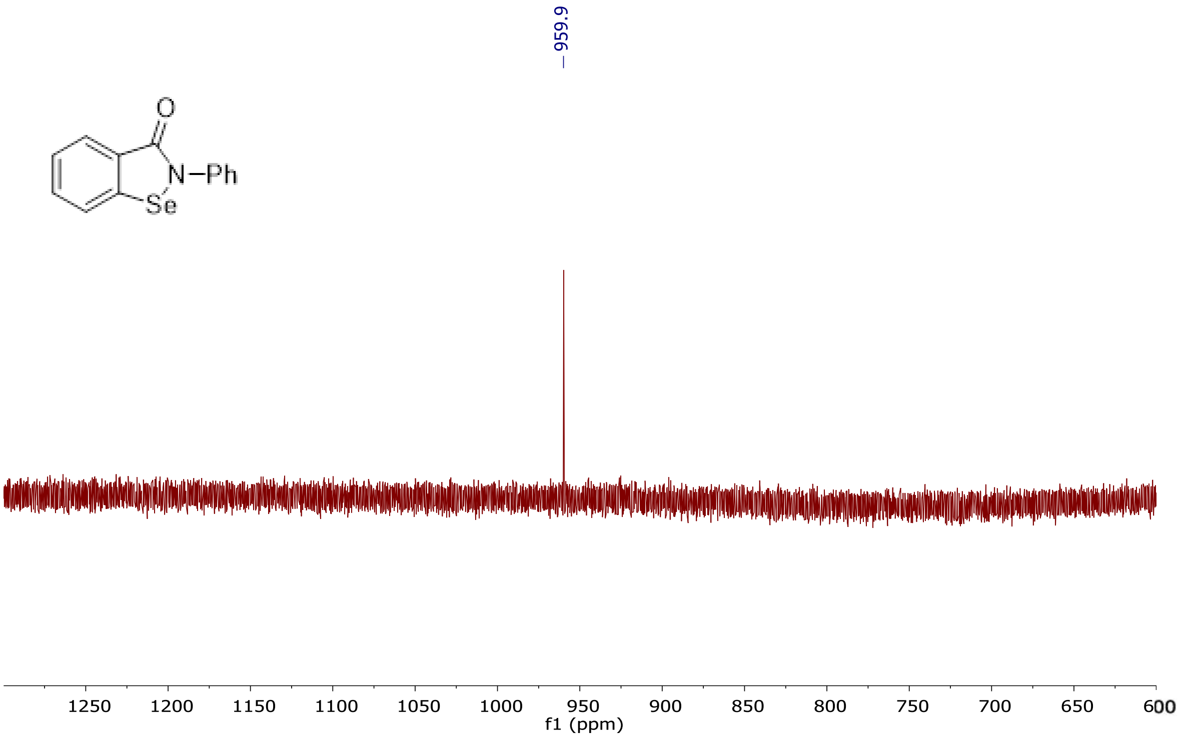

Figure S4. ^{1}H NMR Spectrum of Seleninamide 2 (400 MHz, $CDCl_3$).

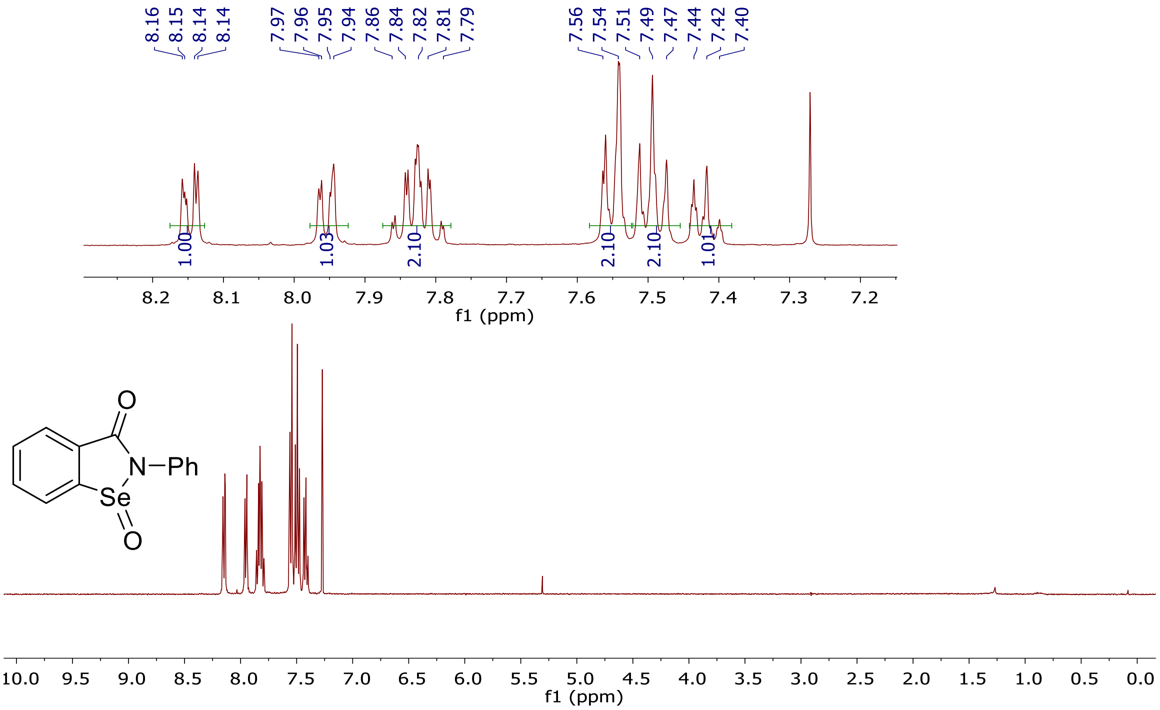

Figure S5. ^{13}**C NMR Spectrum of Seleninamide 2 (101 MHz, CDCl$_3$).**

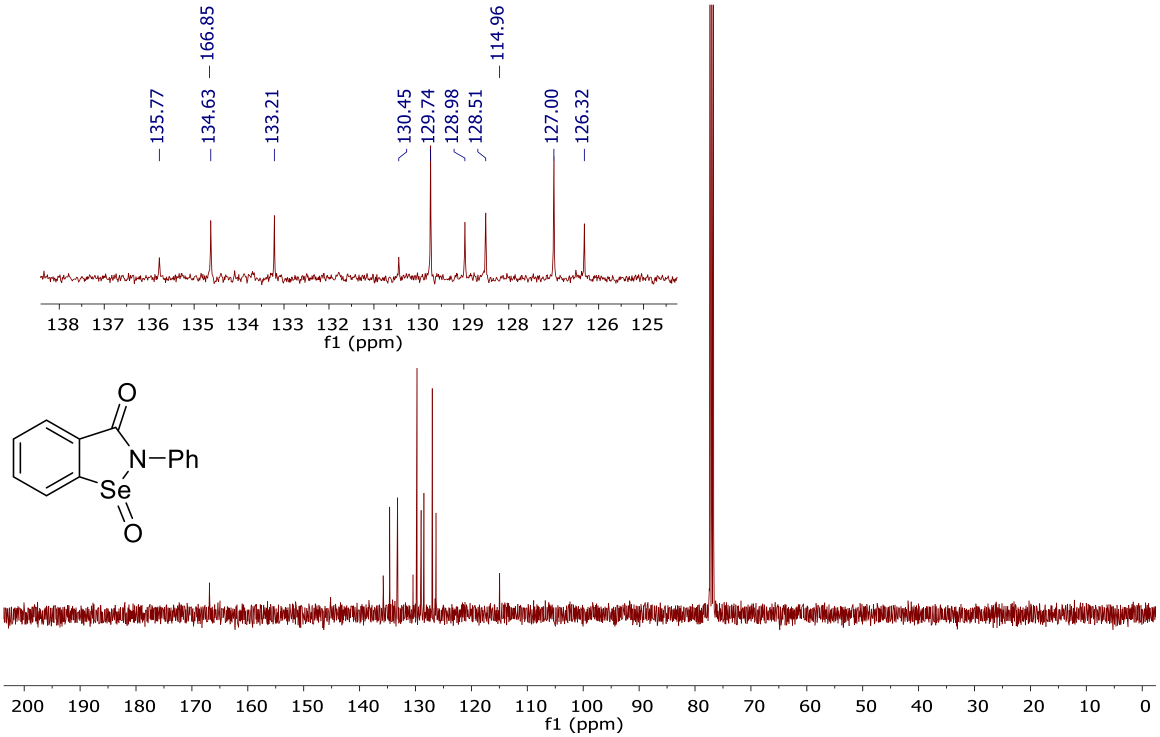

Figure S6. ^{77}**Se NMR Spectrum of Seleninamide 2 (76 MHz, CDCl$_3$).**

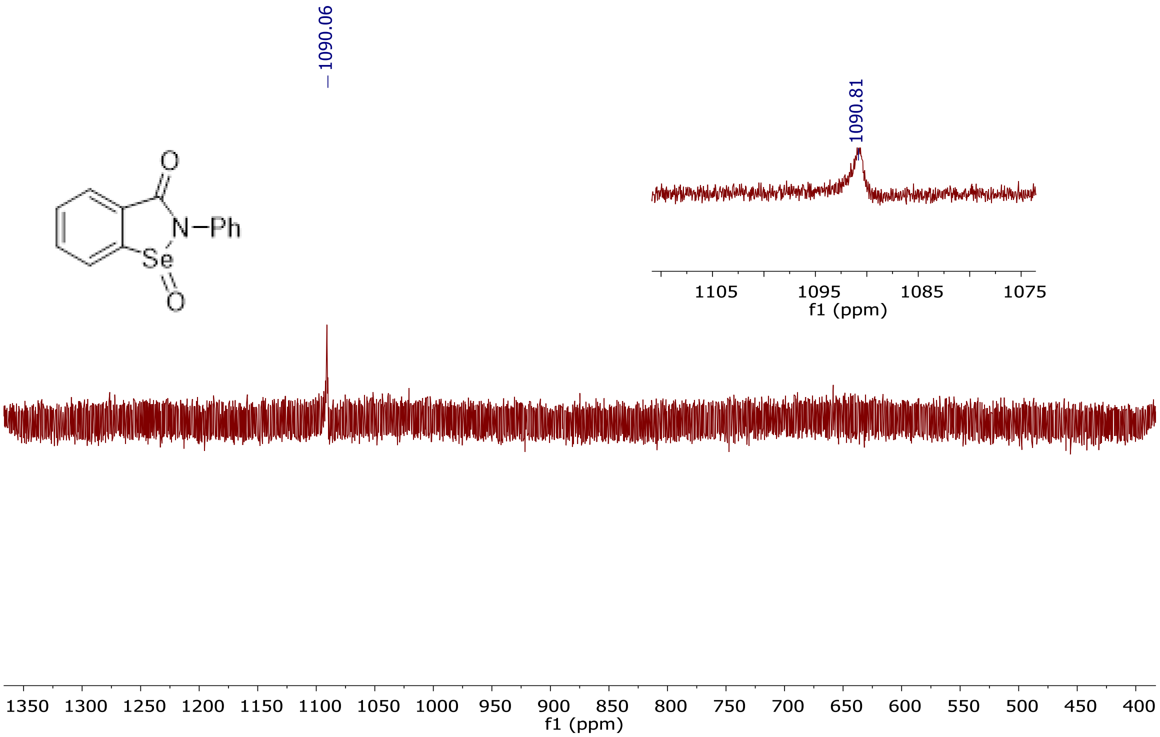

Figure S7. ^{1}H NMR Spectrum of Seleninic Acid 3 (400 MHz, DMSO-d$_6$).

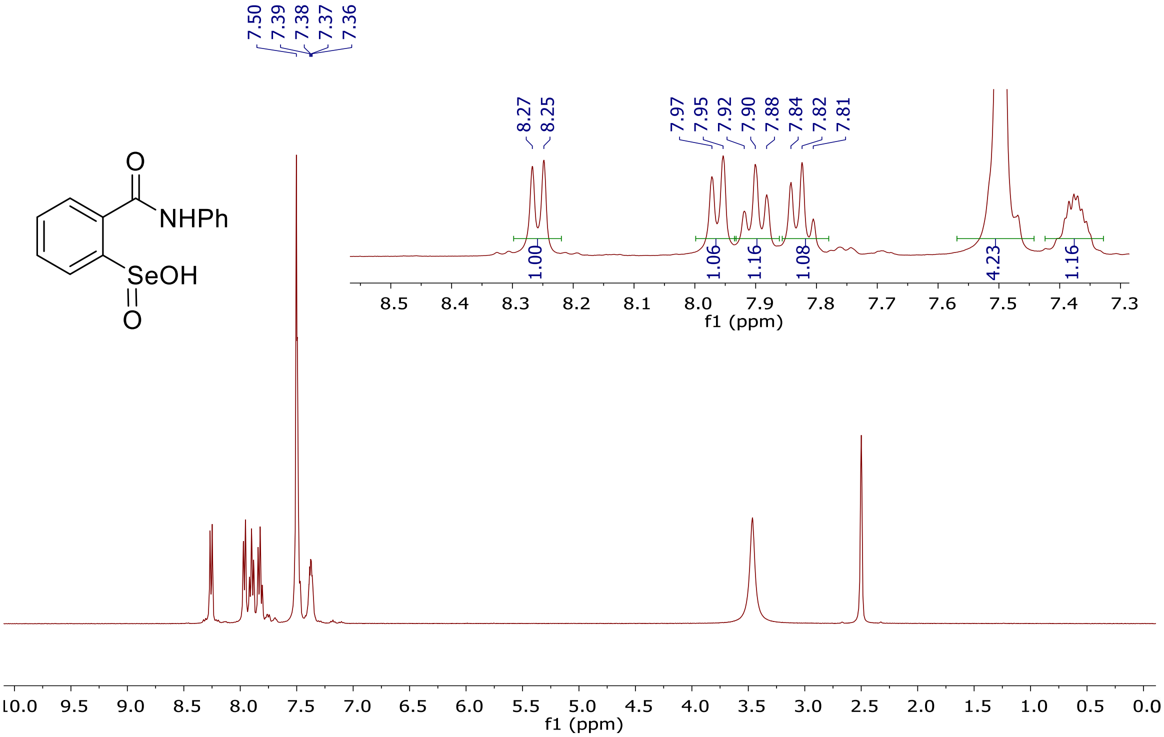

Figure S8. ^{13}C NMR Spectrum of Seleninic Acid 3 (101 MHz, DMSO-d$_6$).

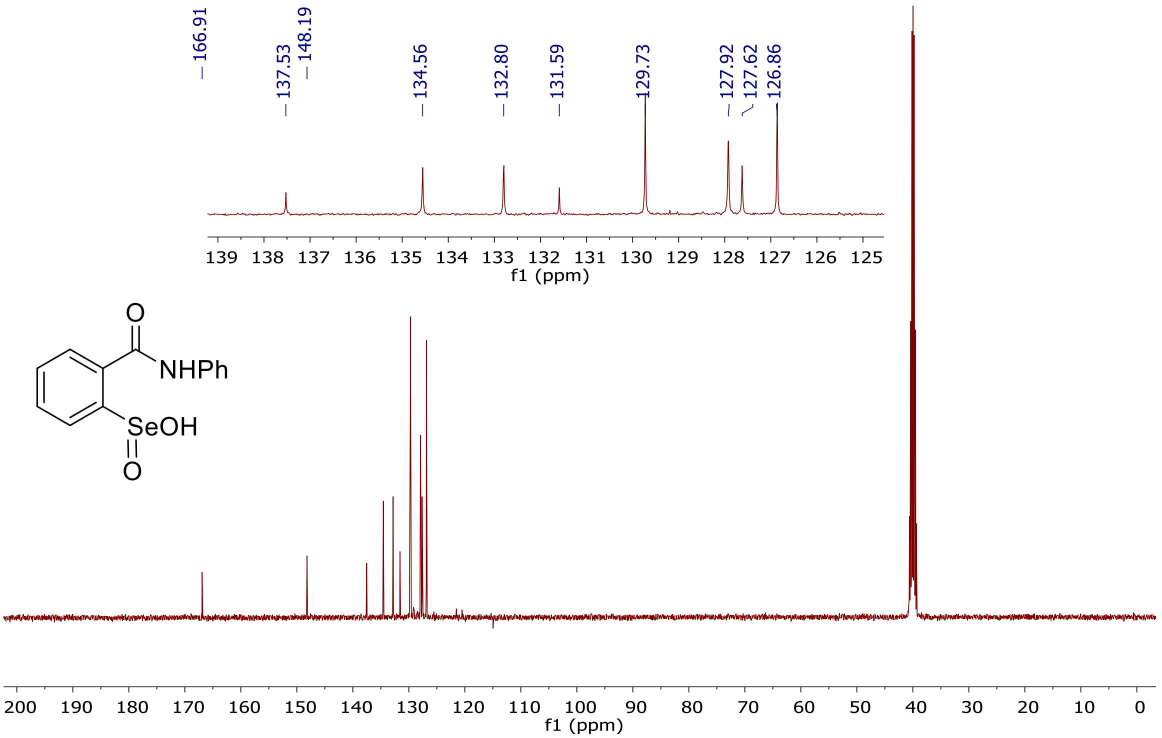

Figure S9. ^{77}Se NMR Specrum of Seleninic Acid 3 (76 MHz, DMSO-d$_6$).

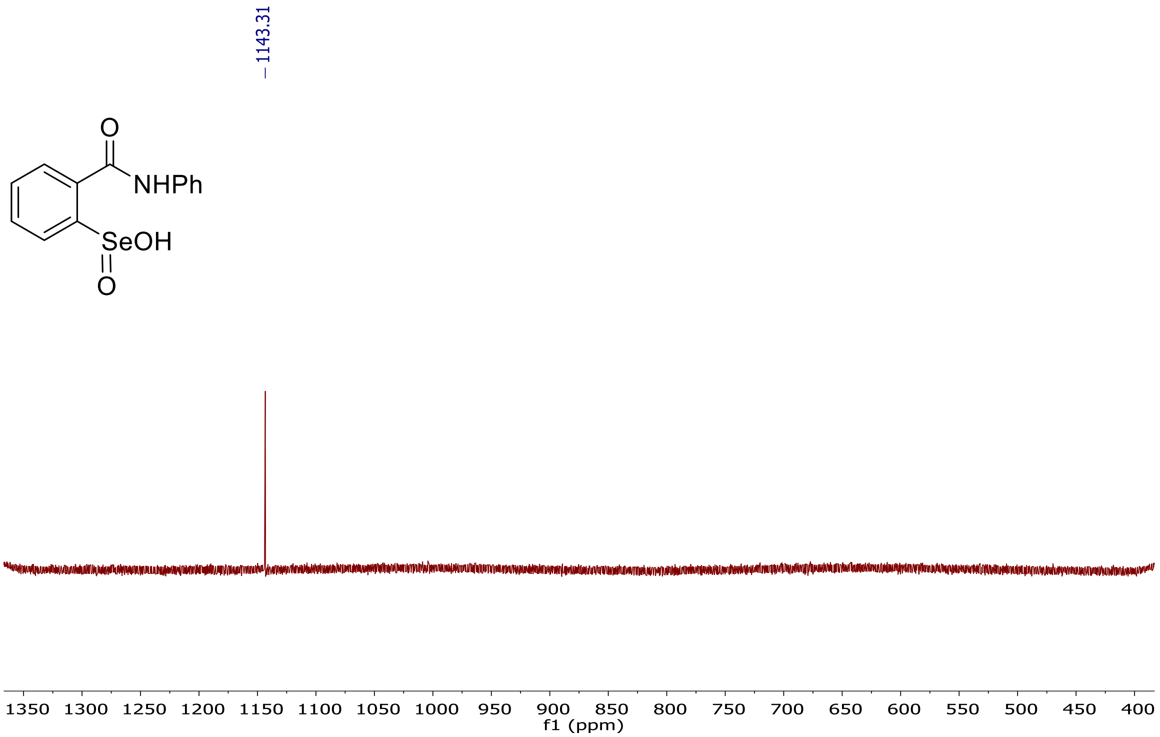

Figure S10. ^{1}H NMR Spectrum of Selenenyl Sulfide 6a (400 MHz, CDCl$_3$).

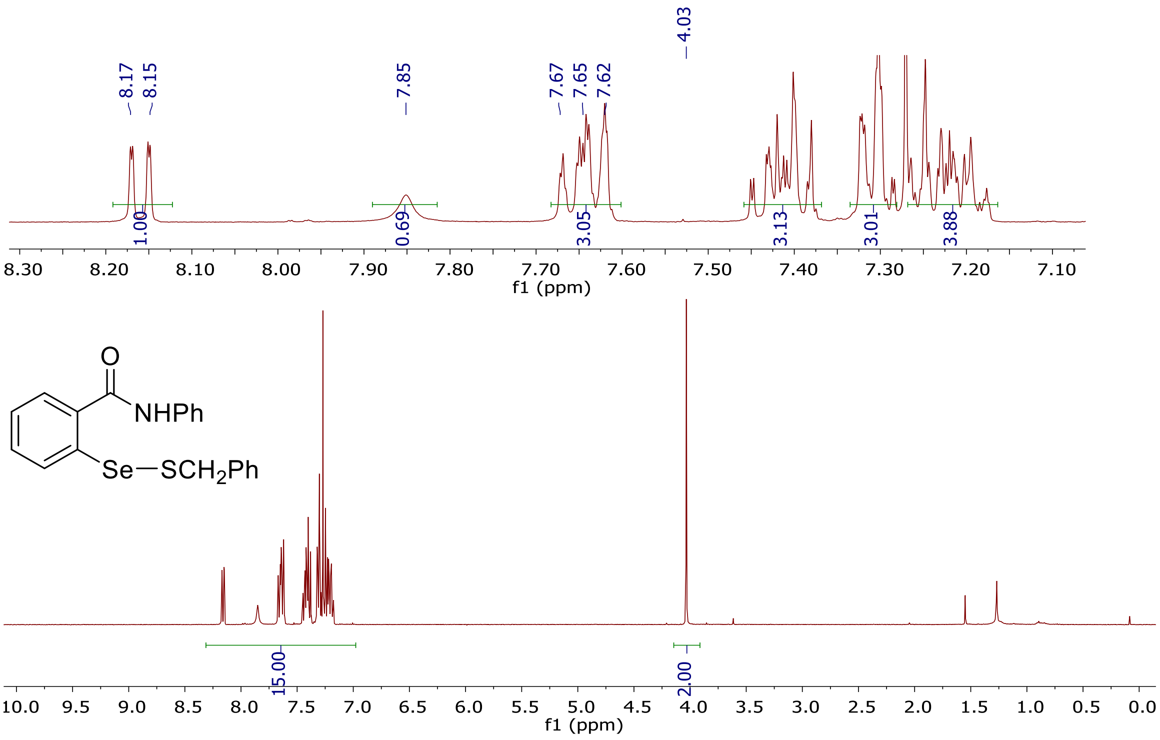

Figure S11. ^{13}C NMR Spectrum of Selenenyl Sulfide 6a (101 MHz, $CDCl_3$).

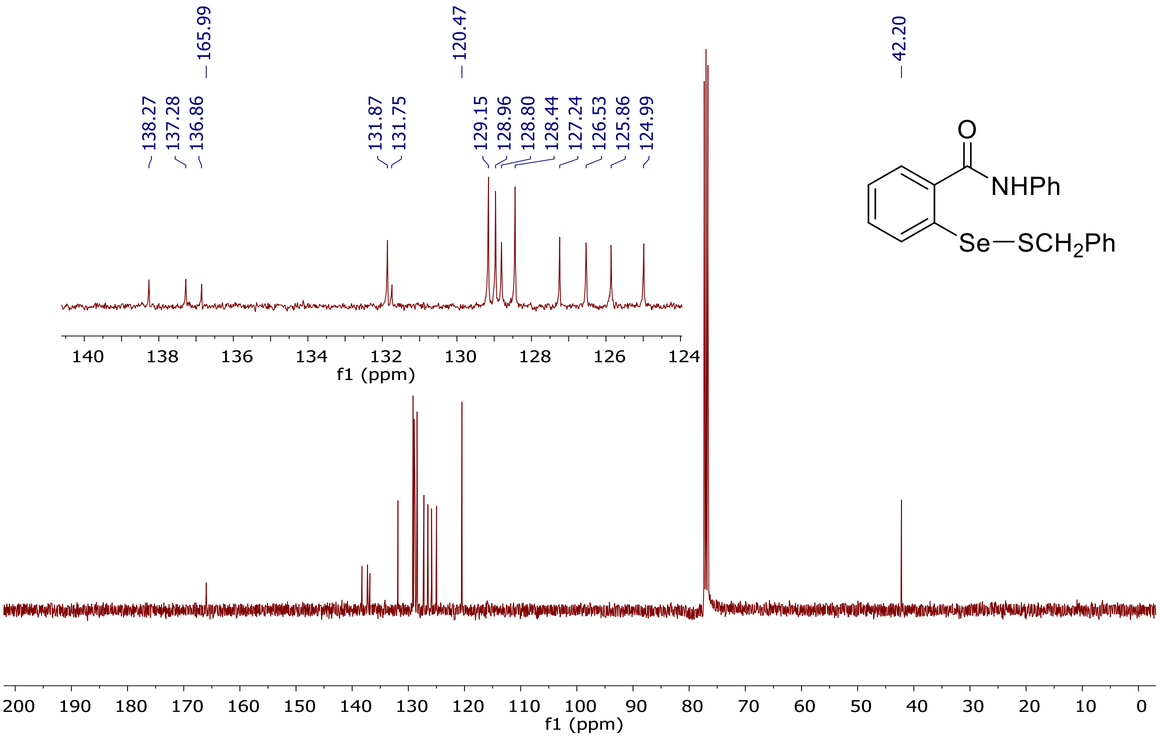

Figure S12. ^{77}Se NMR Spectrum of Selenenyl Sulfide 6a (76 MHz, $CDCl_3$).

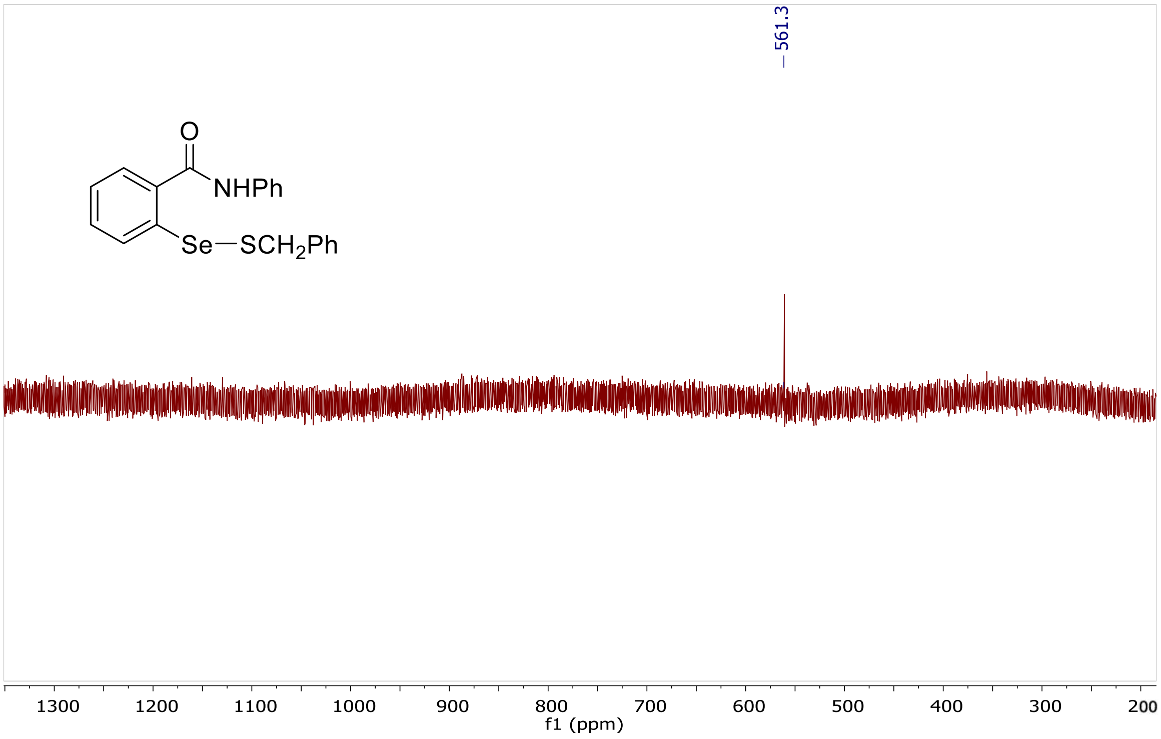

Figure S13. ^{1}H NMR Spectrum of Selenenyl Sulfide 6b (400 MHz, DMSO-d$_6$).

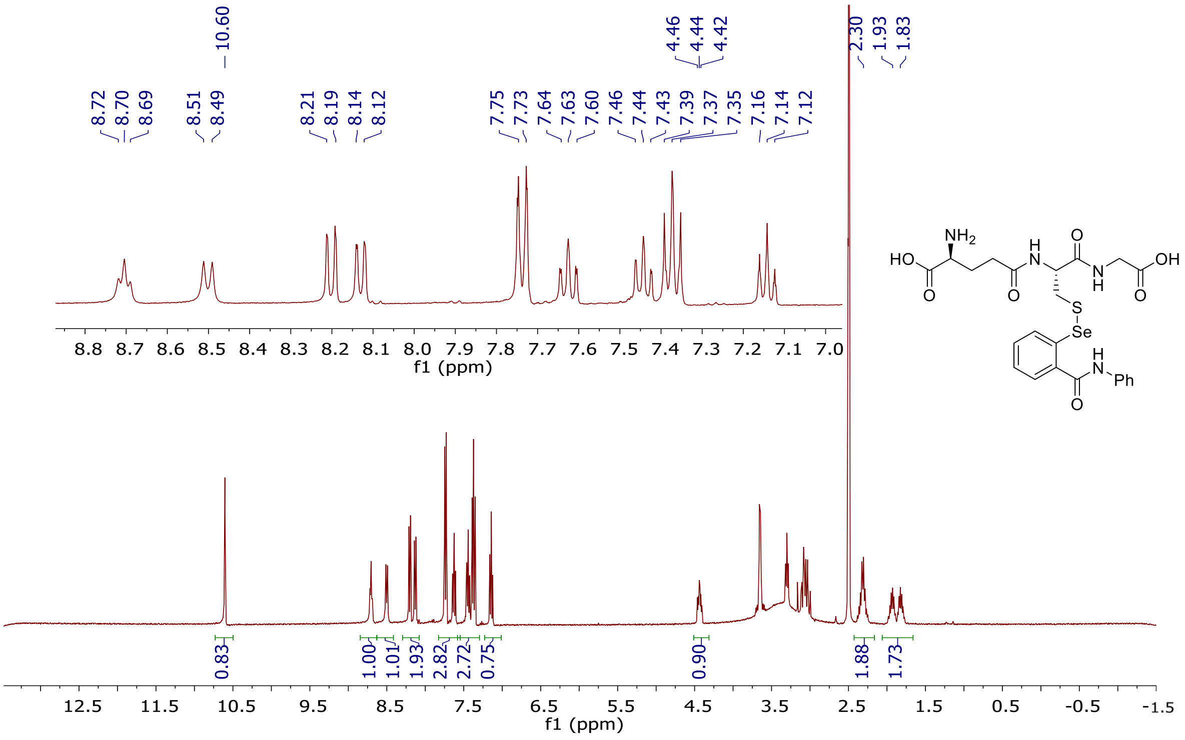

Figure S14. ^{13}C NMR Spectrum of Selenenyl Sulfide 6b (101 MHz, DMSO-d$_6$).

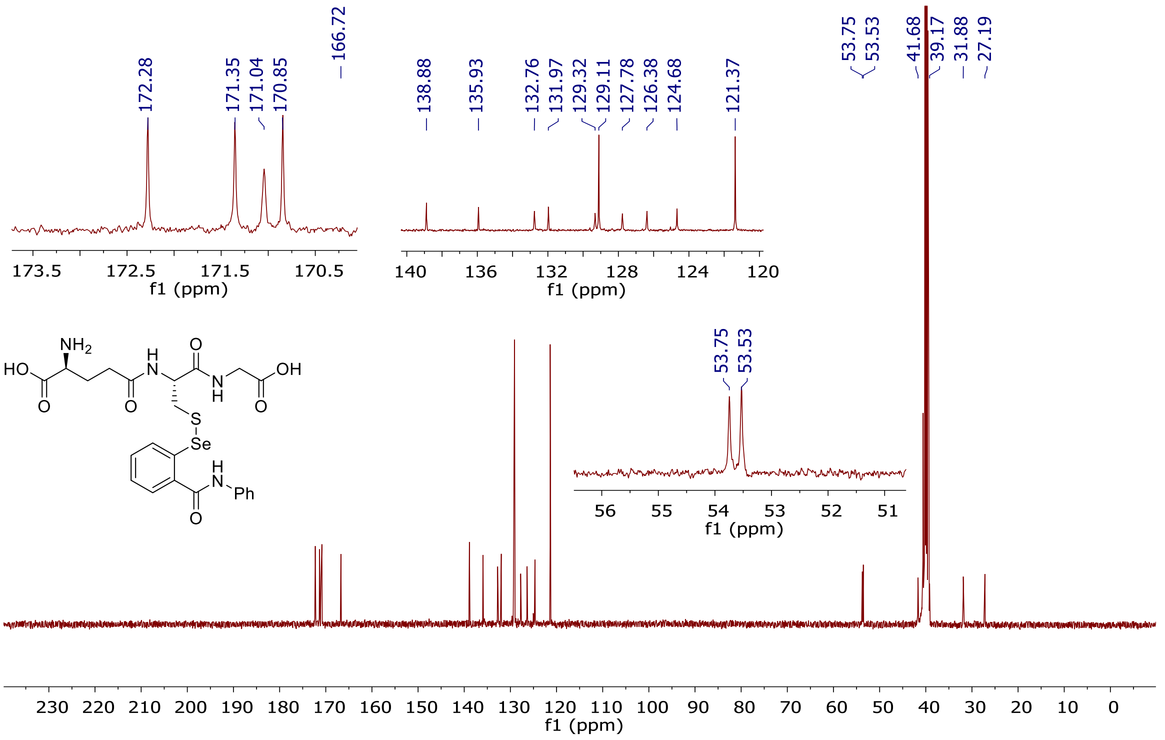

Figure S15. ^{77}Se NMR Spectrum of Selenenyl Sulfide 6b (76 MHz, $CDCl_3$).

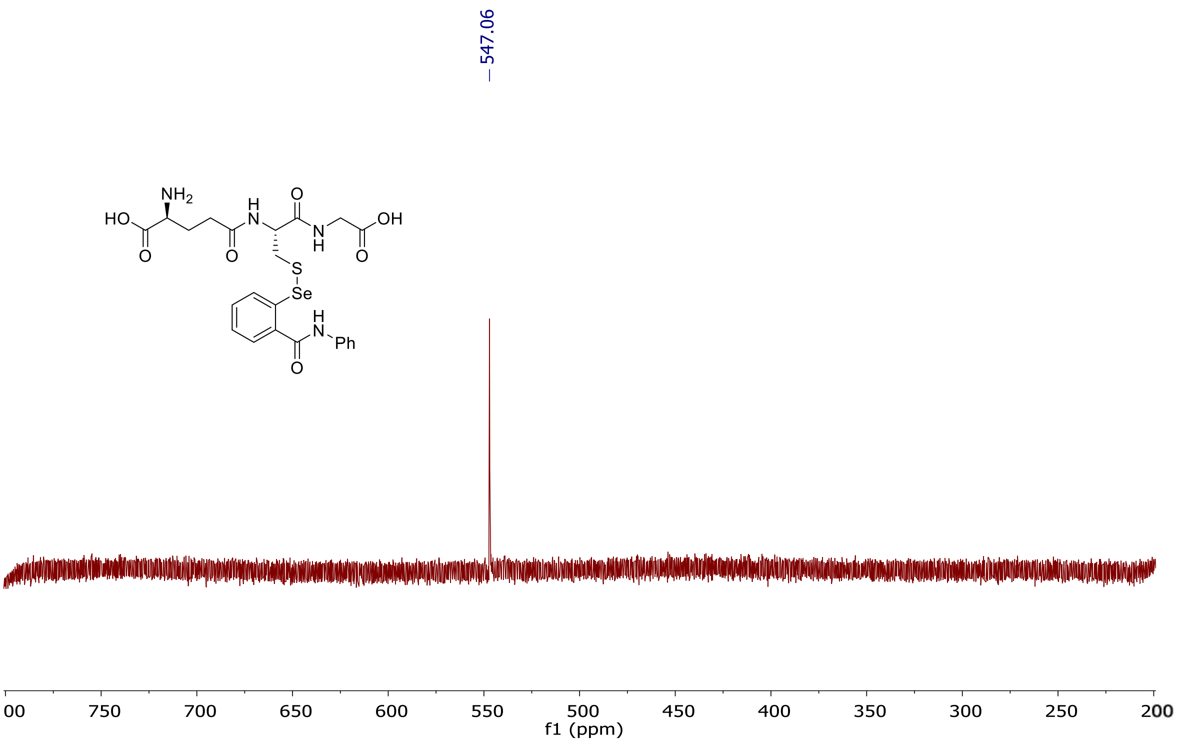

Figure S16. ^{1}H NMR Spectrum of Selenenyl Sulfide 6c (400 MHz, $CDCl_3$).

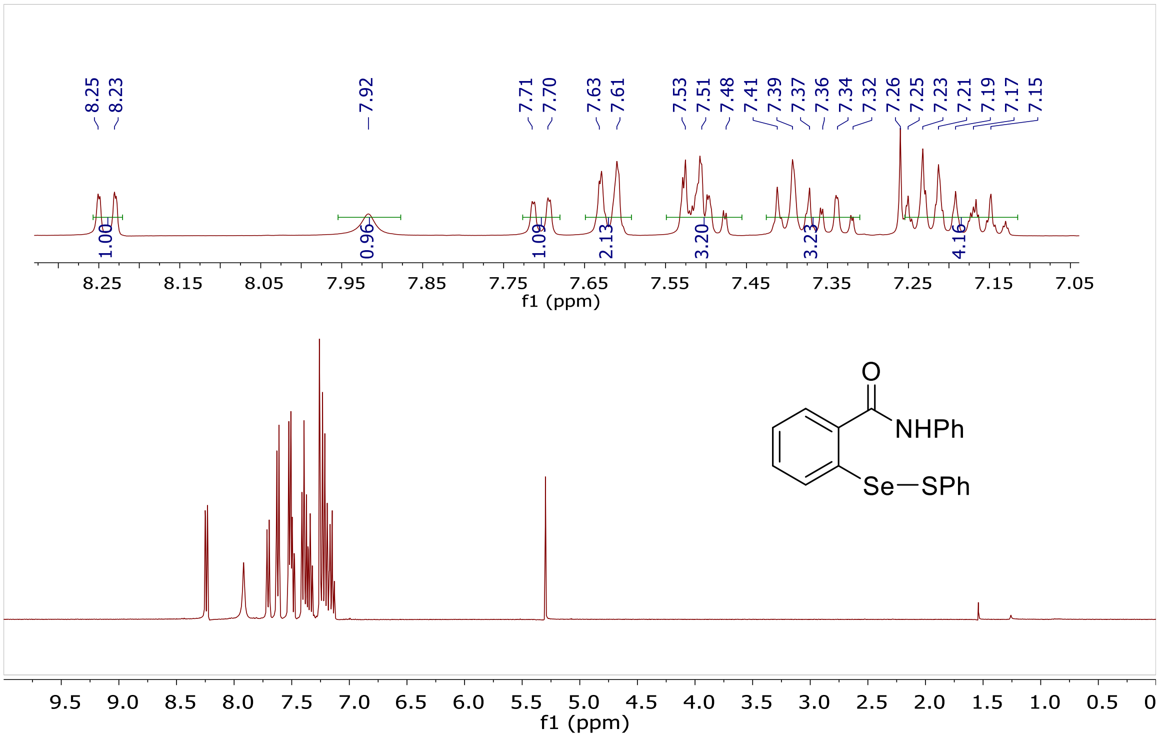

Figure S17. ^{13}C NMR Spectrum of Selenenyl Sulfide 6c (101 MHz, CDCl$_3$).

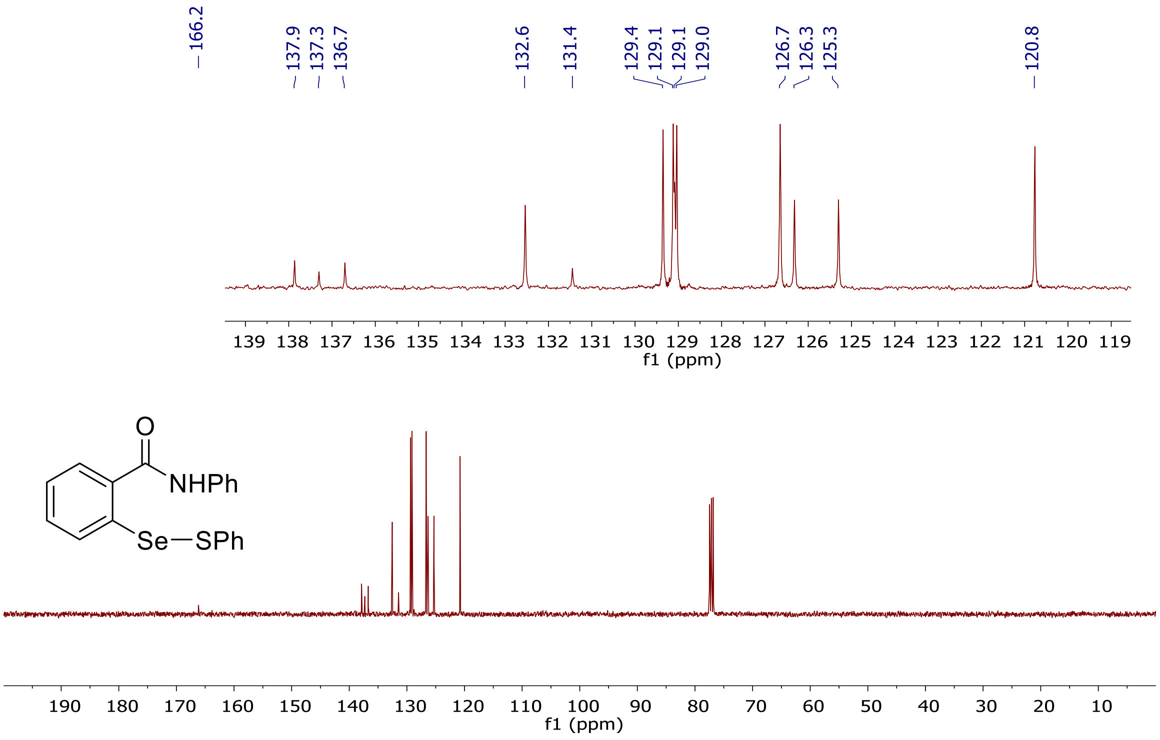

Figure S18. ^{77}Se NMR Spectrum of Selenenyl Sulfide 6c (76 MHz, CDCl$_3$).

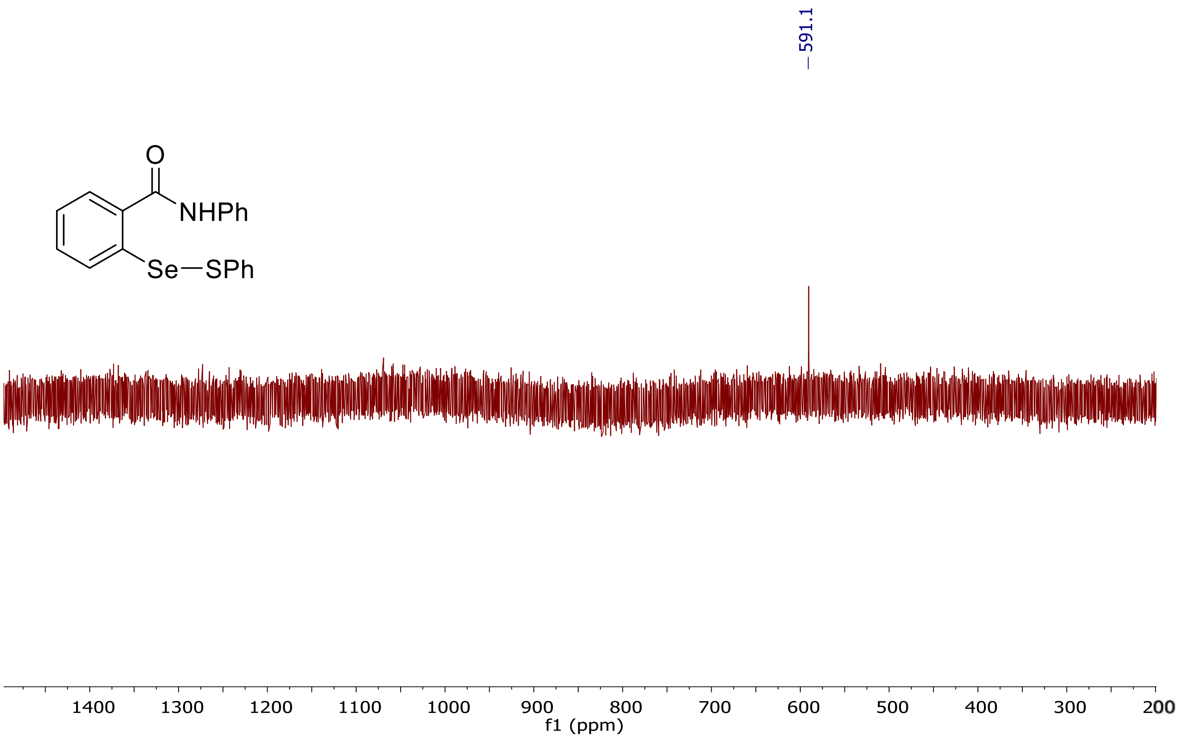

Figure S19. ^{1}H NMR Spectrum of Diselenide 7 (400 MHz, DMSO-d6).

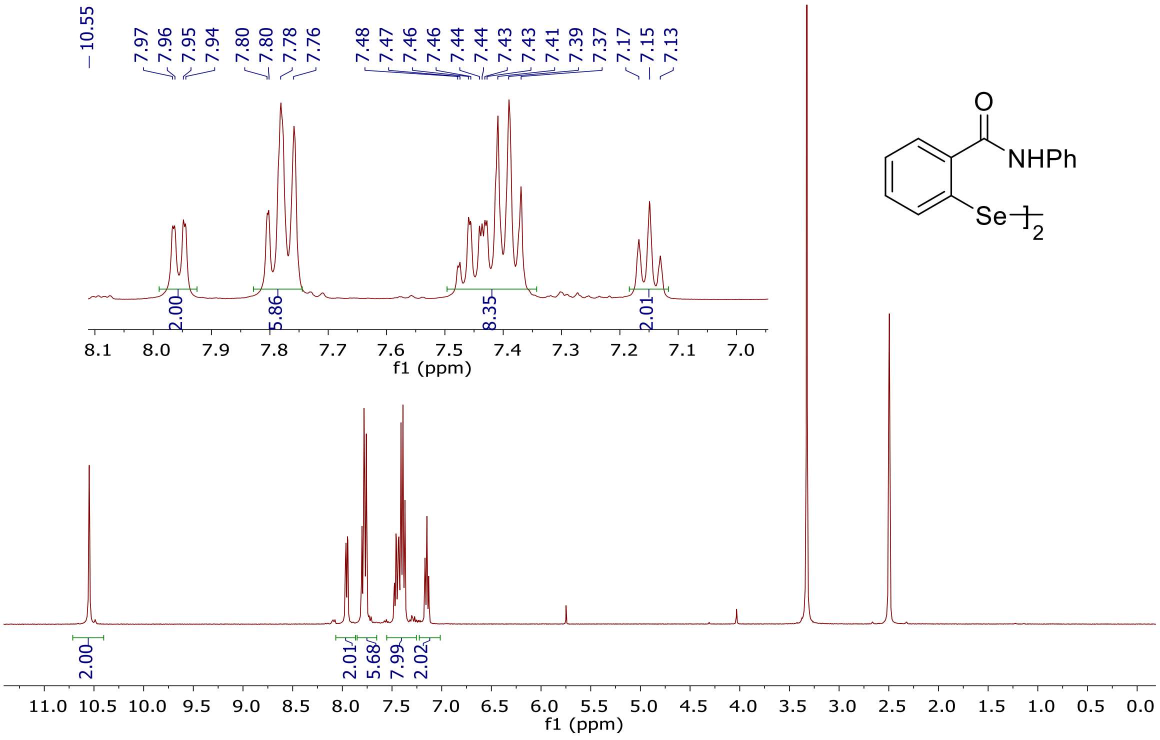

Figure S20. ^{13}C NMR Spectrum of Diselenide 7 (101 MHz, DMSO-d6).

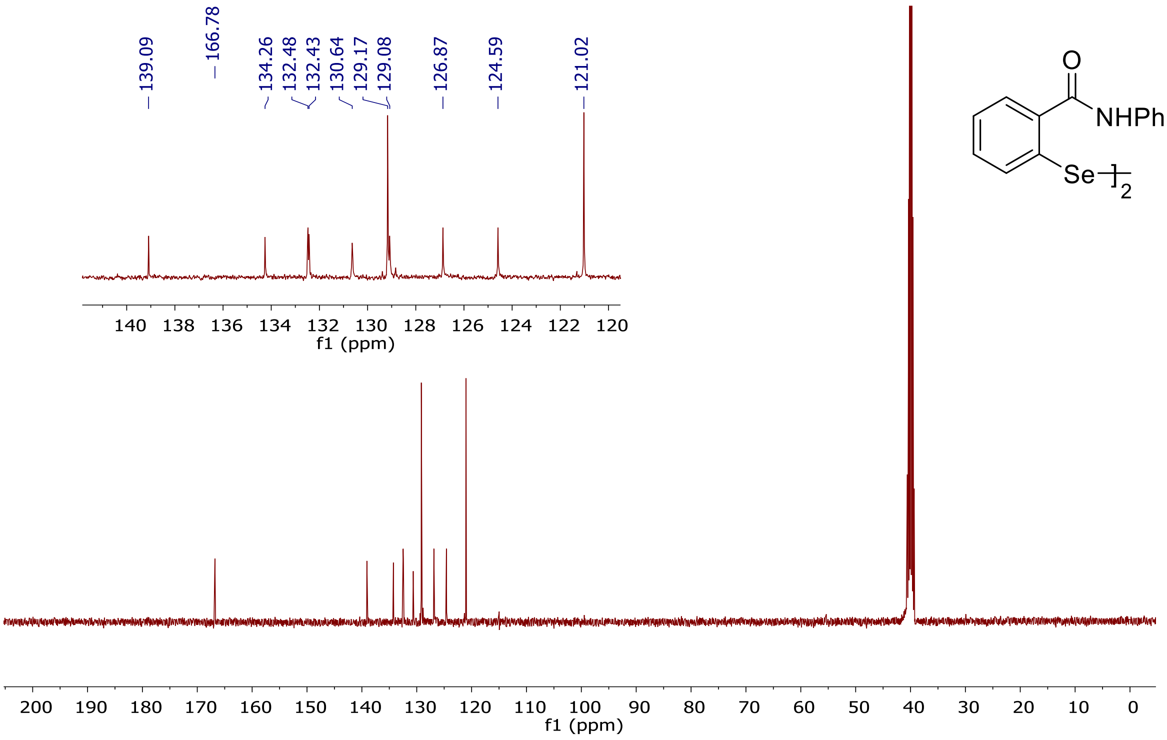

Figure S21. ^{77}Se NMR Spectrum of Diselenide 7 (76 MHz, DMSO-d_6).

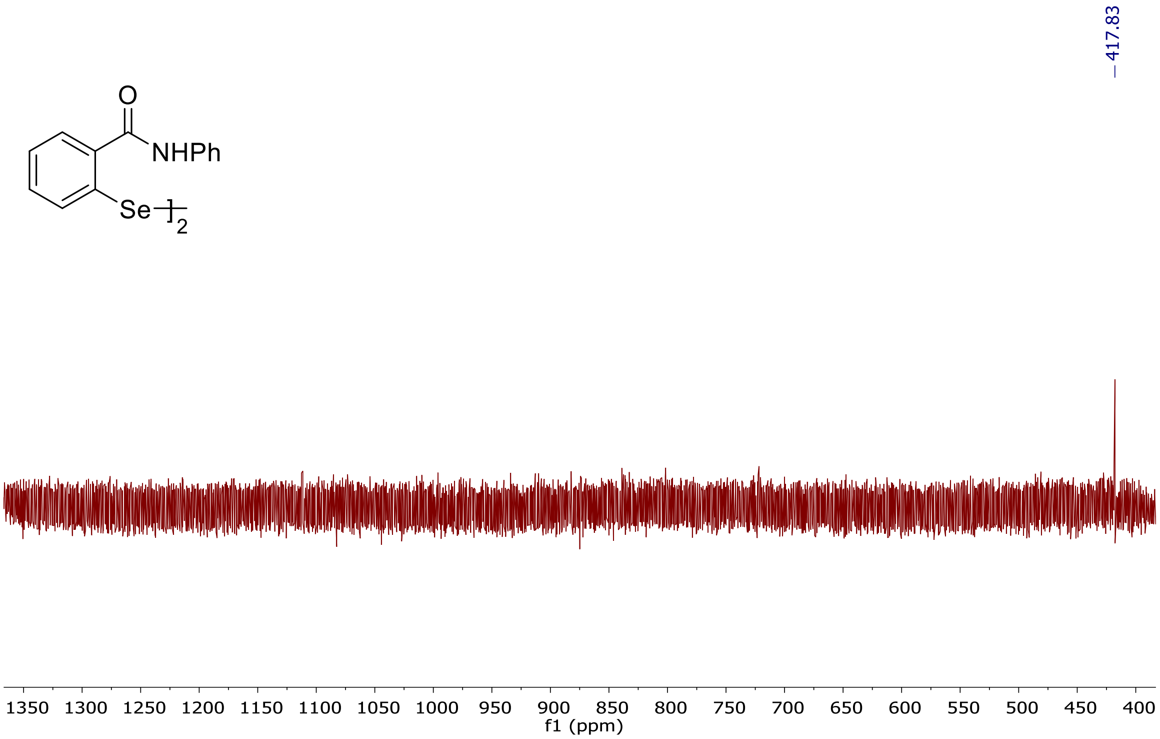

Figure S22. ^{1}H NMR Spectrum of Selenide 22 (400 MHz, CDCl$_3$).

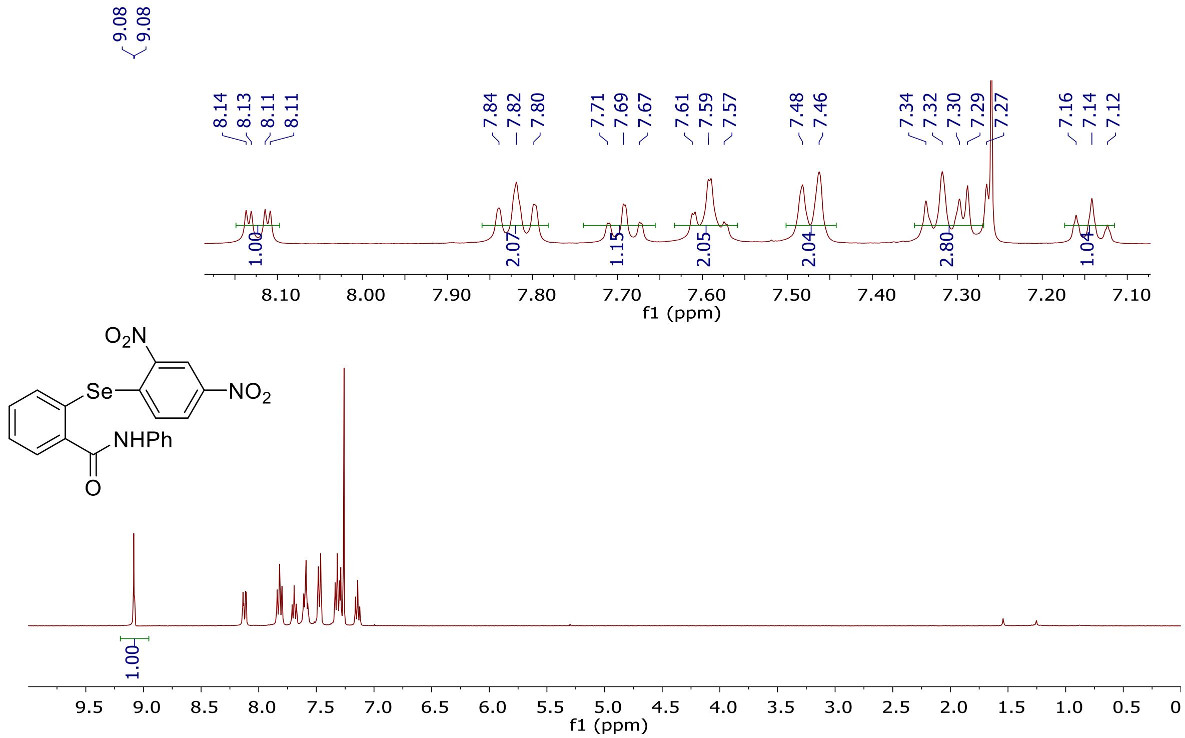

Figure S23. ^{13}C NMR Spectrum of Selenide 22 (101 MHz, $CDCl_3$).

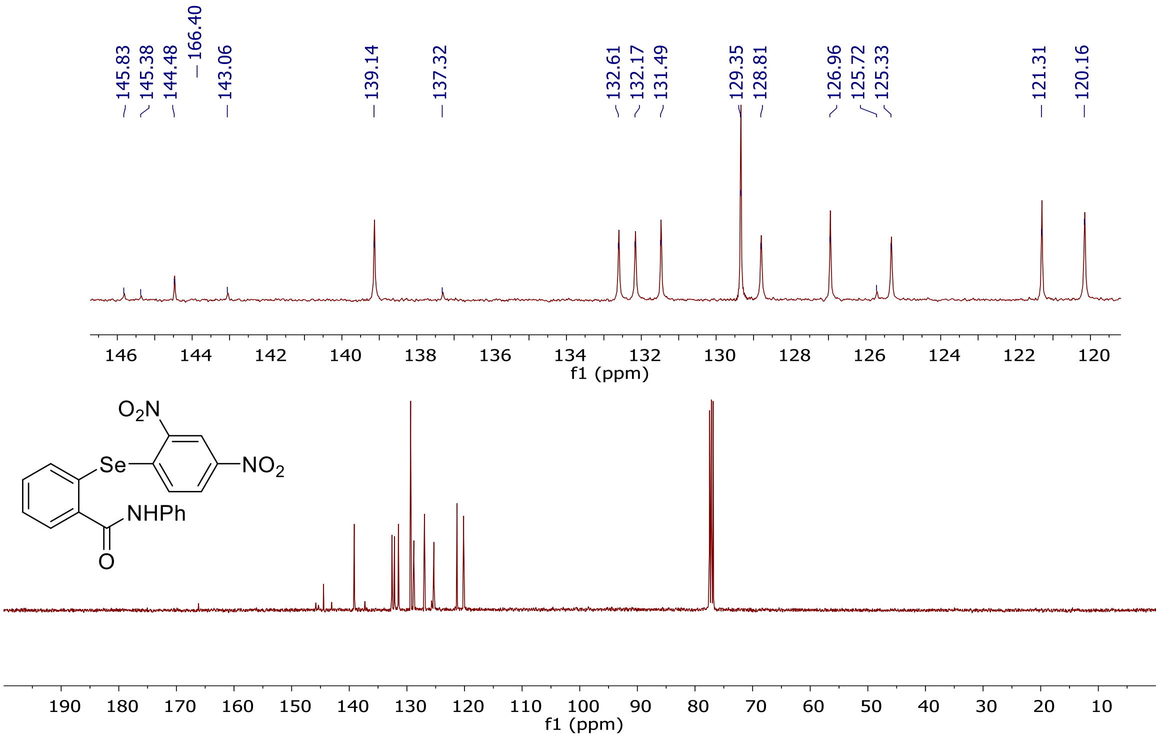

Figure S24. ^{77}Se NMR Spectrum of Selenide 22 (76 MHz, $CDCl_3$).

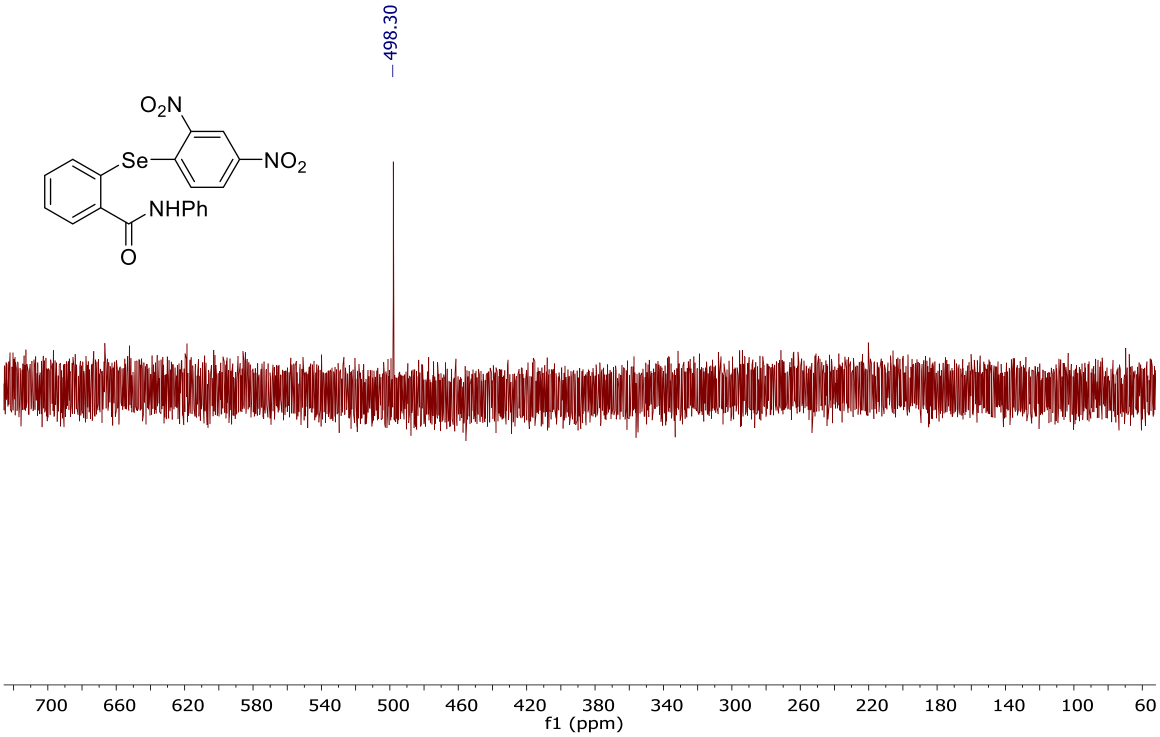

Figure S25. ^{77}Se NMR Spectrum of the Product of Oxidation of Seleninic Acid 3 with 0.5 Equiv of H_2O_2.

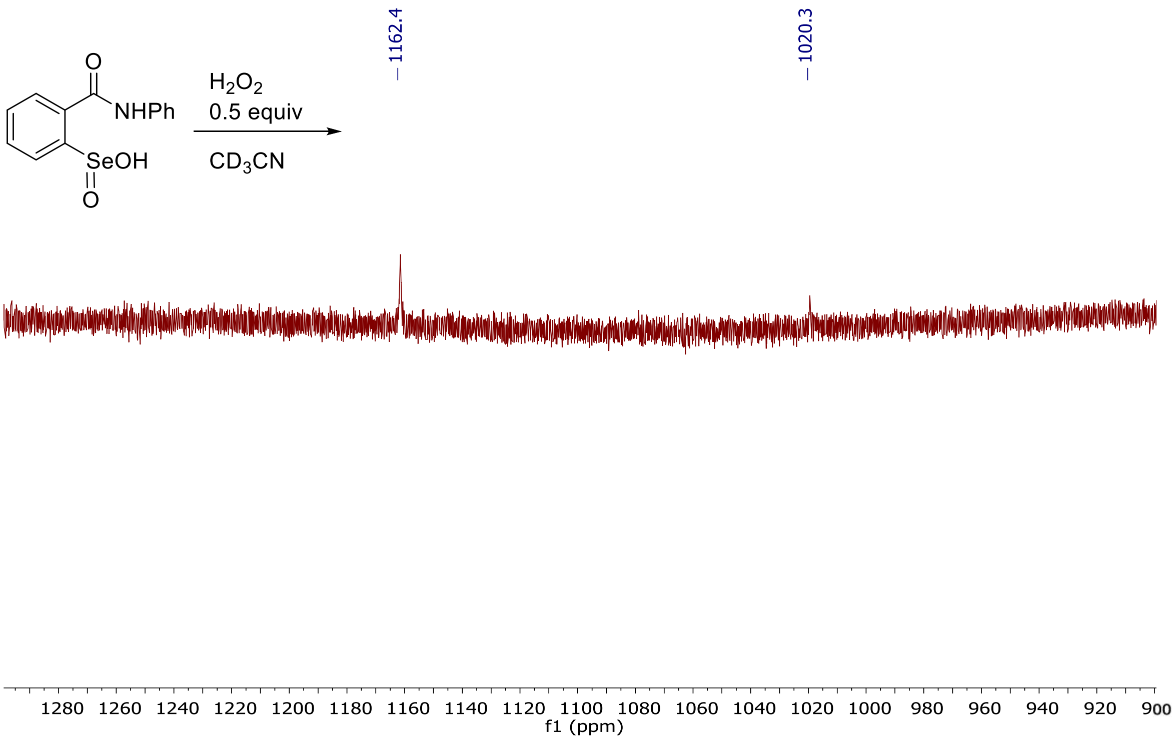

Figure S26. Methanolysis of Seleninamide 2.

NMR Spectroscopy: Methanol was added in increments to a sample of **2** in $CDCl_3$. The ^{1}H NMR spectrum was recorded after each addition. New signals were observed at 8.4 and 7.6-7.7 ppm that do not match those of **1**, **2** or **3** and are attributed to methanolysis products **17a**, **18a** and/or **18b**.

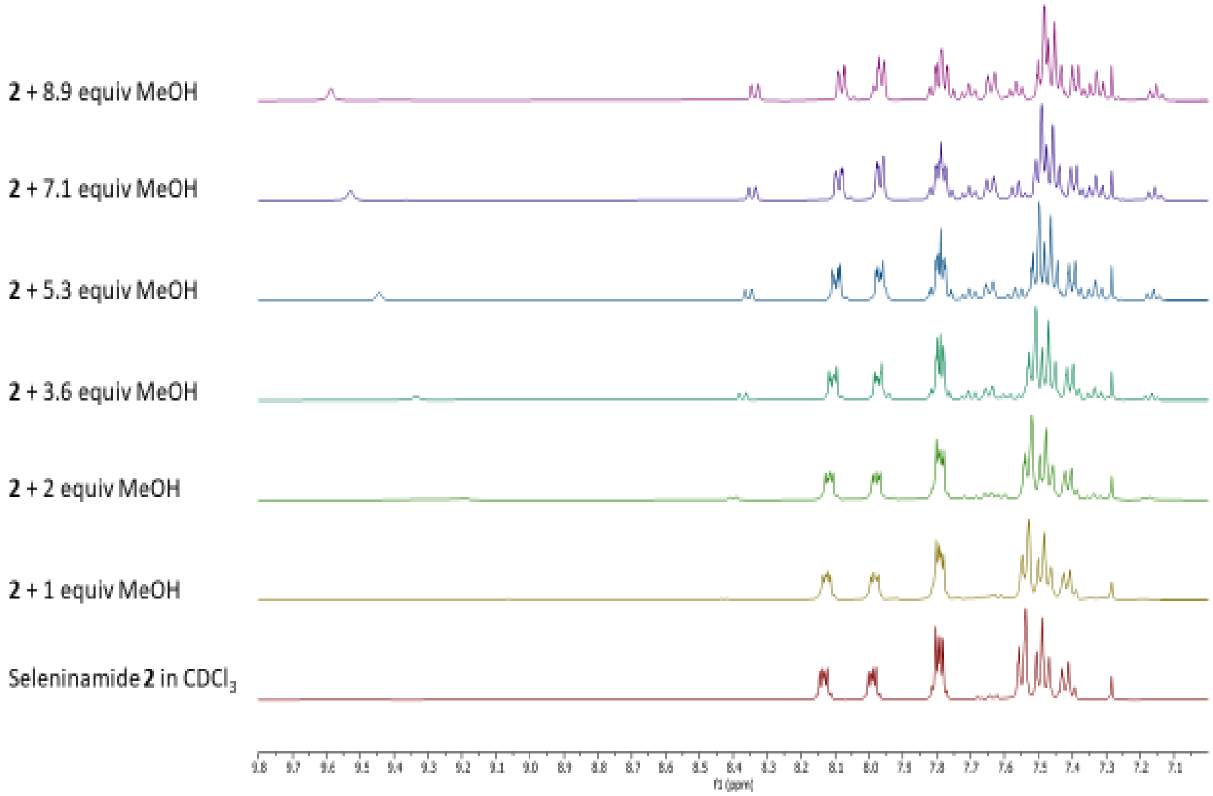

Mass spectrometry: Seleninamide (**2**) (2.2 mg) was dissolved in methanol and analyzed by ESI HRMS immediately (Conditions A) and after standing at room temperature for 1 h (Conditions B). The solution was concentrated in vacuo and the residue was taken up in dry chloroform and analyzed by ESI HRMS (Conditions C). In a similar set of experiments, seleninic acid **3** (10.4 mg) was dissolved in 1 mL of $CHCl_3$/MeOH (95:5) and was analyzed by ESI HRMS immediately (Conditions D) and after standing at room temperature for 1 h (Conditions E). Samples were further

diluted with dry chloroform to the appropriate concentrations for ESI HRMS analysis. The relative abundances of the $[M+H]^+$ peaks under the various conditions are shown below.

Figure S27. Relative abundance of the $[M+H]^+$ peaks in the methanolysis of 2.

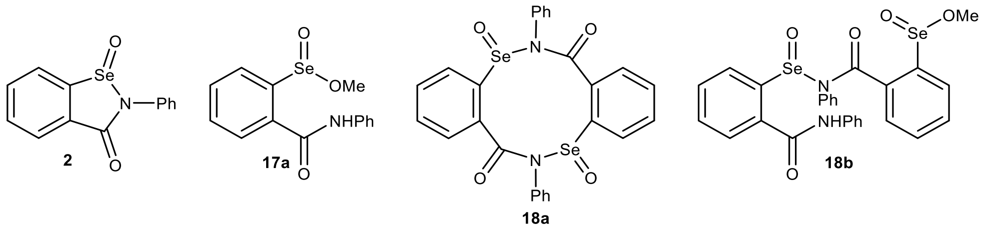

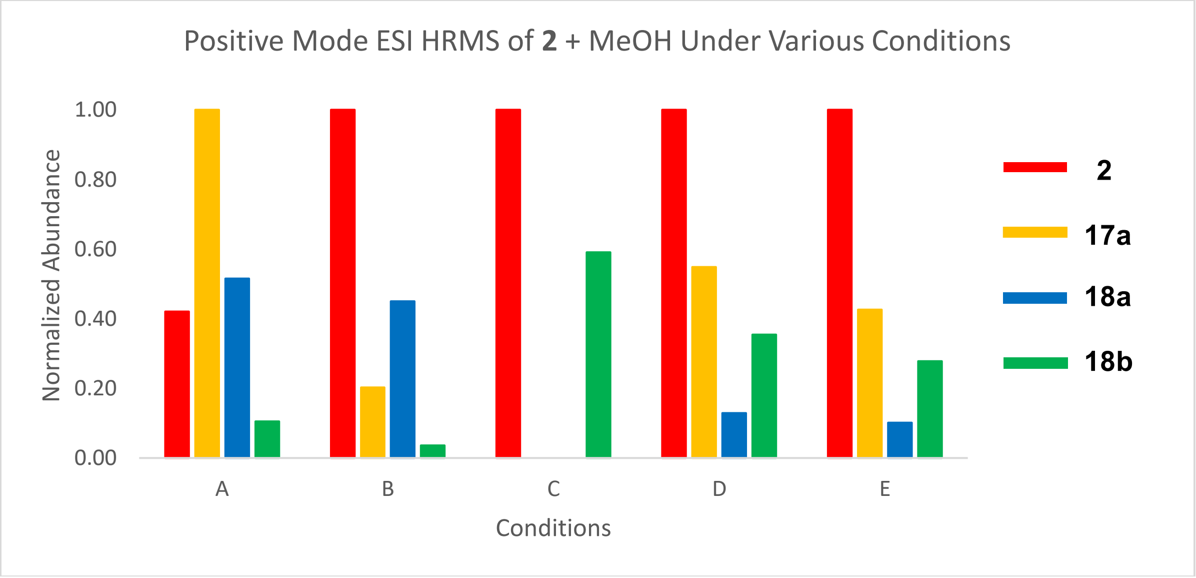

Footnote: Seleninamide (**32**) (2.2 mg) was dissolved in methanol and analyzed by ESI HRMS immediately (Conditions A) and after standing at room temperature for 1 h (Conditions B). The solution was concentrated in vacuo and the residue was taken up in dry chloroform and analyzed by ESI HRMS (Conditions C). In a similar set of experiments, seleninamide **32** (10.4 mg) was dissolved in 1 mL of $CHCl_3$/MeOH (95:5) and was analyzed by ESI HRMS immediately (Conditions D) and after standing at room temperature for 1 h (Conditions E). Samples were further diluted with dry chloroform to the appropriate concentrations for ESI HRMS analysis.

Figure S28. Oxidation of Selenenyl Sulfide 6a with Hydrogen Peroxide.

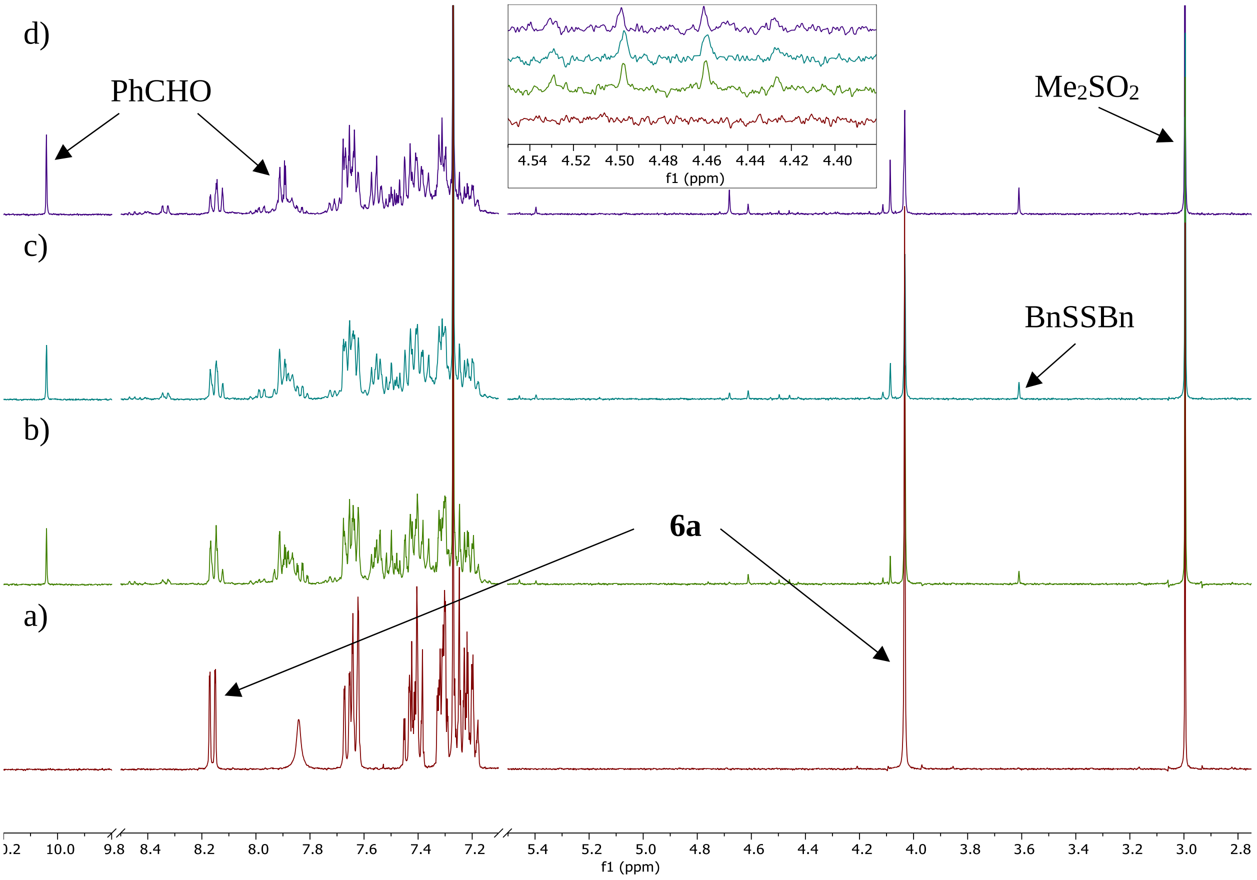

Footnote: ^{1}H NMR spectra of the oxidation of selenenyl sulfide **6a** with 1 equiv of hydrogen peroxide in $CDCl_3$ at a) 0 min; b) 25 min; c) 45 min ($t_{1/2}$); d) 100 min. Insert: Expansion of the region from 4.55-3.38 ppm showing the faint AB quartet from transient thiolseleninate **19.**

Figure S29. Molecular Modelling of 14 and 15.

Ebselen selenonic acid **14** (anti)

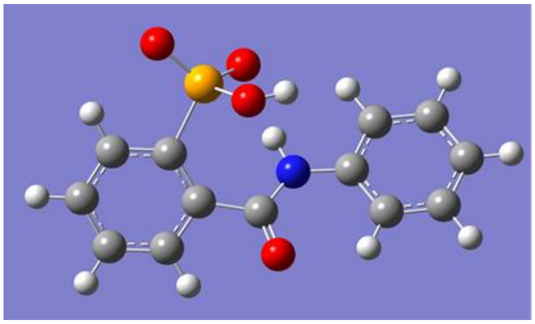

Ebselen selenonic acid **14** (syn)

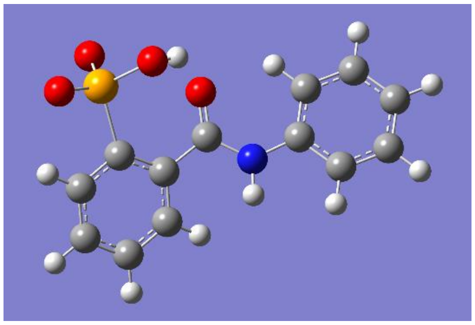

Ebselen peroxyseleninic acid **15** (syn)

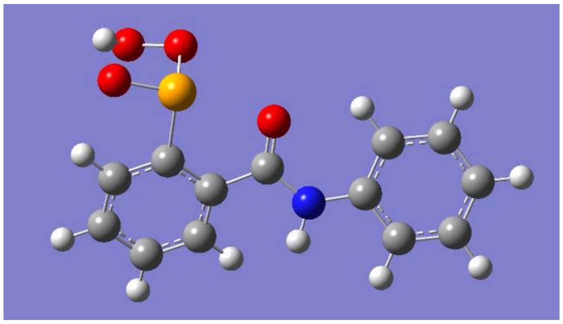

Ebselen peroxyseleninic acid **15 (**anti)

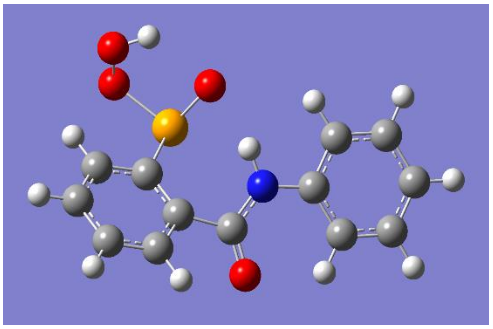

Ebselen selenonic acid **14** (anti)

E= -3259.43032274 a.u.

```
Symbolic Z-matrix:
 Charge =  0 Multiplicity = 1
 C                    -1.68731   1.32106   2.73114
 C                    -1.80348   2.56229   2.11788
 C                    -2.14023   2.63877   0.77427
 C                    -2.38895   1.4799    0.05327
 C                    -2.27578   0.22092   0.64678
 C                    -1.89631   0.16511   1.99218
 H                    -1.44573   1.24462   3.78171
 H                    -1.62946   3.46152   2.69237
 H                    -2.21962   3.60006   0.28582
 H                    -2.66469   1.54799  -0.98872
 Se                   -1.56389  -1.46081   3.03334
 O                    -2.07869  -1.14034   4.51807
 O                    -0.06832  -1.98384   2.78786
```

```
 C                    -2.47679  -1.01469  -0.17948
 O                    -3.28845  -1.03734  -1.09674
 N                    -1.78338  -2.1449    0.149
 O                    -2.70846  -2.63437   2.39185
 H                    -2.34388  -2.89872   1.52548
 C                    -2.45898  -3.81413  -0.93077
 C                    -1.85238  -5.05699  -0.74493
 C                    -3.51421  -3.6812   -1.83319
 C                    -2.30088  -6.16594  -1.46208
 H                    -1.02061  -5.16182  -0.03327
 C                    -3.96338  -4.7908   -2.54975
 H                    -3.99248  -2.70181  -1.97988
 C                    -3.35668  -6.03301  -2.36435
 H                    -1.82329  -7.14558  -1.31557
 H                    -4.79541  -4.68581  -3.26097
 H                    -3.71043  -6.90754  -2.92933
 H                    -0.91779  -2.25167   0.63824
```

Ebselen selenonic acid **14** (syn)

E= -3259.42711479 a.u.

```
Symbolic Z-matrix:
 Charge =  0 Multiplicity = 1
 C                     2.72106   1.43572  -1.70267
 C                     3.50049   1.40191  -0.5511
 C                     2.88336   1.31819   0.69327
 C                     1.49708   1.2681    0.78677
 C                     0.67932   1.33403  -0.34751
 C                     1.33149   1.41128  -1.58992
 H                     3.17348   1.4661   -2.68552
 H                     4.58037   1.42666  -0.63296
 H                     3.48153   1.27895   1.59606
 H                     1.01013   1.15962   1.74725
 Se                    0.43705   1.48756  -3.31145
 O                     1.52824   1.26607  -4.4594
 O                    -0.56226   2.75911  -3.38913
 C                    -0.81      1.20581  -0.06032
 O                    -1.59743   1.37284  -0.97611
 N                    -1.15733   0.86058   1.21343
 O                    -0.59501   0.05587  -3.24926
 H                    -1.4788    0.35604  -2.98407
 C                    -2.45712   0.72764   1.76001
 C                    -3.63429   0.8669    1.014
 C                    -2.53413   0.44383   3.13234
 C                    -4.86489   0.71909   1.65036
 H                    -3.5758    1.08409  -0.04056
 C                    -3.77053   0.29829   3.74843
 H                    -1.621     0.3459    3.70942
```

```
 C                    -4.94535   0.43474   3.01066
 H                    -5.77152   0.82739   1.06559
 H                    -3.81326   0.07795   4.80901
 H                    -5.90991   0.32065   3.49118
 H                    -0.40948   0.69968   1.8819
```

Ebselen peroxyseleninic acid **15** (syn)

E = -3259.38500138 a.u.

```
Symbolic Z-matrix:
 Charge =  0 Multiplicity = 1
 C                    -2.63783   1.82924   0.61083
 C                    -2.62349   3.05054   1.28127
 C                    -1.48679   3.44148   1.98336
 C                    -0.36083   2.62317   2.00233
 C                    -0.35133   1.39925   1.32173
 C                    -1.51778   1.01096   0.64491
 H                    -3.50594   1.49029   0.05724
 H                    -3.50041   3.68732   1.26453
 H                    -1.4759    4.38009   2.5249
 H                     0.49451   2.93778   2.59009
 Se                   -1.69313  -0.72362  -0.298
 O                    -3.19898  -0.55608  -0.93087
 O                    -2.12249  -1.7592    1.1919
 O                    -3.16812  -1.08209   1.90966
 H                    -3.97181  -1.44932   1.48394
 C                     0.82348   0.46536   1.30433
 O                     0.67941  -0.69726   0.93931
 N                     2.0239    0.99342   1.68667
 H                     2.04327   1.98825   1.84581
 C                     5.83816  -0.74731   2.09113
 C                     4.74589  -1.55619   1.78984
 C                     3.4685   -1.02148   1.64097
 C                     3.28209   0.3571    1.79885
 C                     4.37993   1.17366   2.10315
 C                     5.64723   0.6242    2.24742
 H                     6.82591  -1.17833   2.20299
 H                     4.88165  -2.62464   1.66645
 H                     2.62701  -1.65193   1.40212
 H                     4.23787   2.24323   2.22762
 H                     6.4852    1.27034   2.4824
```

Ebselen peroxyseleninic acid **15 (**anti)

E = -3259.38061802

```
Symbolic Z-matrix:
 Charge =  0 Multiplicity = 1
```

C	-1.83209	1.79494	0.00209
C	-2.7296	1.72164	1.06528
C	-2.32371	2.10227	2.3413
C	-1.03015	2.57105	2.55275
C	-0.11893	2.66614	1.49354
C	-0.53954	2.24945	0.22118
H	-2.11142	1.50369	-1.00394
H	-3.7369	1.35773	0.89895
H	-3.00964	2.02772	3.17696
H	-0.7327	2.82282	3.56477
Se	0.65158	2.2458	-1.36305
O	1.39708	3.69545	-1.16578
O	-0.62838	2.75112	-2.62119
O	-1.3301	3.89194	-2.09859
H	-0.78103	4.63319	-2.43188
C	1.29113	3.15601	1.64974
O	1.6983	3.51719	2.74949
N	2.06493	3.1462	0.52405
H	1.66097	2.71317	-0.29123
C	6.03475	4.38462	-0.14615
C	5.44575	4.61801	1.0935
C	4.13806	4.21956	1.35999
C	3.40346	3.57242	0.35914
C	3.99375	3.33704	-0.89018
C	5.29923	3.74028	-1.13899
H	7.05346	4.69999	-0.33776
H	6.00758	5.11891	1.87369
H	3.68873	4.39794	2.32379
H	3.42561	2.83653	-1.66888
H	5.74032	3.54988	-2.11063

Reference for Molecular Modelling:

Gaussian 09, Revision A.02,
M. J. Frisch, G. W. Trucks, H. B. Schlegel, G. E. Scuseria,
M. A. Robb, J. R. Cheeseman, G. Scalmani, V. Barone, B. Mennucci,
G. A. Petersson, H. Nakatsuji, M. Caricato, X. Li, H. P. Hratchian,
A. F. Izmaylov, J. Bloino, G. Zheng, J. L. Sonnenberg, M. Hada,
M. Ehara, K. Toyota, R. Fukuda, J. Hasegawa, M. Ishida, T. Nakajima,
Y. Honda, O. Kitao, H. Nakai, T. Vreven, J. A. Montgomery, Jr.,
J. E. Peralta, F. Ogliaro, M. Bearpark, J. J. Heyd, E. Brothers,
K. N. Kudin, V. N. Staroverov, R. Kobayashi, J. Normand,
K. Raghavachari, A. Rendell, J. C. Burant, S. S. Iyengar, J. Tomasi,
M. Cossi, N. Rega, J. M. Millam, M. Klene, J. E. Knox, J. B. Cross,
V. Bakken, C. Adamo, J. Jaramillo, R. Gomperts, R. E. Stratmann,
O. Yazyev, A. J. Austin, R. Cammi, C. Pomelli, J. W. Ochterski,
R. L. Martin, K. Morokuma, V. G. Zakrzewski, G. A. Voth,
P. Salvador, J. J. Dannenberg, S. Dapprich, A. D. Daniels,
O. Farkas, J. B. Foresman, J. V. Ortiz, J. Cioslowski,
and D. J. Fox, Gaussian, Inc., Wallingford CT, 2009.
